# Cross-modal sensory compensation increases mosquito attraction to humans

**DOI:** 10.1126/sciadv.adn5758

**Published:** 2025-01-01

**Authors:** Takeshi Morita, Nia G. Lyn, Ricarda K. von Heynitz, Olivia V. Goldman, Trevor R. Sorrells, Matthew DeGennaro, Benjamin J. Matthews, Leah Houri-Zeevi, Leslie B. Vosshall

**Affiliations:** ^1^ Laboratory of Neurogenetics and Behavior, The Rockefeller University, New York, NY 10065, USA.; ^2^ Howard Hughes Medical Institute, New York, NY 10065, USA.; ^3^ Kavli Neural Systems Institute, New York, NY 10065, USA.

## Abstract

Sensory compensation occurs when loss of one sense leads to enhanced perception by another sense. We have identified a previously undescribed mechanism of sensory compensation in female *Aedes aegypti* mosquitoes. Odorant receptor co-receptor (*Orco*) mutants show enhanced attraction to human skin temperature and increased heat-evoked neuronal activity in foreleg sensory neurons. *Ir140*, a foreleg-enriched member of the ionotropic receptor (IR) superfamily of sensory receptors, is up-regulated in *Orco* mutant legs. *Ir140*, *Orco* double mutants do not show the enhanced heat seeking seen in *Orco* single mutants, suggesting that up-regulation of *Ir140* in the foreleg is a key mechanism underlying sensory compensation in *Orco* mutants. Because *Orco* expression is sparse in legs, this sensory compensation requires an indirect, long-range mechanism. Our findings highlight how female *Aedes aegypti* mosquitoes, despite suffering olfactory sensory loss, maintain the overall effectiveness of their host-seeking behavior by up-regulating attraction to human skin temperature, further enhancing their status as the most dangerous predator of humans.

## Teaser

Mosquitoes enhance sensitivity to human body heat by a sensory compensation mechanism when their sense of smell is disrupted.

## INTRODUCTION

Animals are endowed with diverse sensory modalities to take in information from their environment, encompassing thermal, chemical, auditory, mechanical, visual, and other cues. These senses work together to form a representation in the brain of the sensory richness of the external world to guide appropriate behaviors. Because of the importance of multisensory integration, animals have mechanisms to adapt when they experience a loss or impairment in one of their sensory modalities, either through acute injury, chronic disease, or a congenital loss from birth. In this way, loss of vision, hearing, or touch leads to the development of a heightened acuity in the remaining senses. This adaptive process, termed sensory compensation, involves the reorganization and reallocation of neural resources to amplify the function of intact sensory modalities. For example, blind individuals often exhibit superior auditory and tactile perception as they adapt to prioritize these senses to navigate their environment safely and effectively (*1*, *2*). Similarly, people with hearing loss may develop a more acute sense of vision or touch to compensate for their auditory impairment (*3*). Sensory compensation highlights the power of neural plasticity and the capacity to adapt to changing sensory inputs, enabling individuals to navigate their world despite sensory impairment.

Sensory compensation is observed throughout the animal kingdom but is best understood at a mechanistic level in mammals and involves anatomical reorganization at thalamic and primary sensory cortex levels. These changes expand and refine connections within neighboring primary sensory cortical areas. For example, classical experiments in the ferret visual system revealed that eliminating retinal axon projection in one hemisphere led to alternative terminal space for these axons within the auditory thalamus (*4*). These changes were subsequently reflected in changes in cortical representation (*5*, *6*). Functional reorganization also occurs between primary sensory cortical regions from different sensory modalities. Braille reading by blind individuals activates the primary visual cortex, suggesting an expansion of somatosensory function into brain regions generally dedicated to visual processing (*7*). Moreover, in congenitally blind individuals, the occipital lobe, typically associated with visual processing, is activated during auditory localization tasks (*8*).

Sensory compensation has also been observed by modulating existing neuronal circuits without requiring major anatomical rewiring. For example, *Caenorhabditis elegans* nematodes deprived of their sense of touch exhibit enhanced olfactory-mediated behavioral performance (*9*). This enhancement was attributed to the strengthening of synaptic transmission in the olfactory circuit, resulting from reduced neuropeptide signaling caused by impaired mechanosensory circuits (*9*). Similarly, studies in visually deprived rats revealed strengthening of AMPA receptor–mediated synaptic transmission in pyramidal neurons of layer 2/3 somatosensory barrel cortex that were dependent on long-distance serotonin signaling from the raphe nuclei (*10*, *11*). *Drosophila melanogaster* flies with olfactory loss showed enhanced sensitivity to sugar, mediated by an elevated sugar response in protocerebral anterior medial dopaminergic neurons in the mushroom body (*12*). These anatomical and functional studies highlight the diverse mechanisms that each sensory system and organism uses while also emphasizing the shared necessity of sensory compensation. For organisms that depend heavily on integrating various sensory cues, this adaptive process is critical for guiding vital behavioral outputs.

**Fig. 1.**
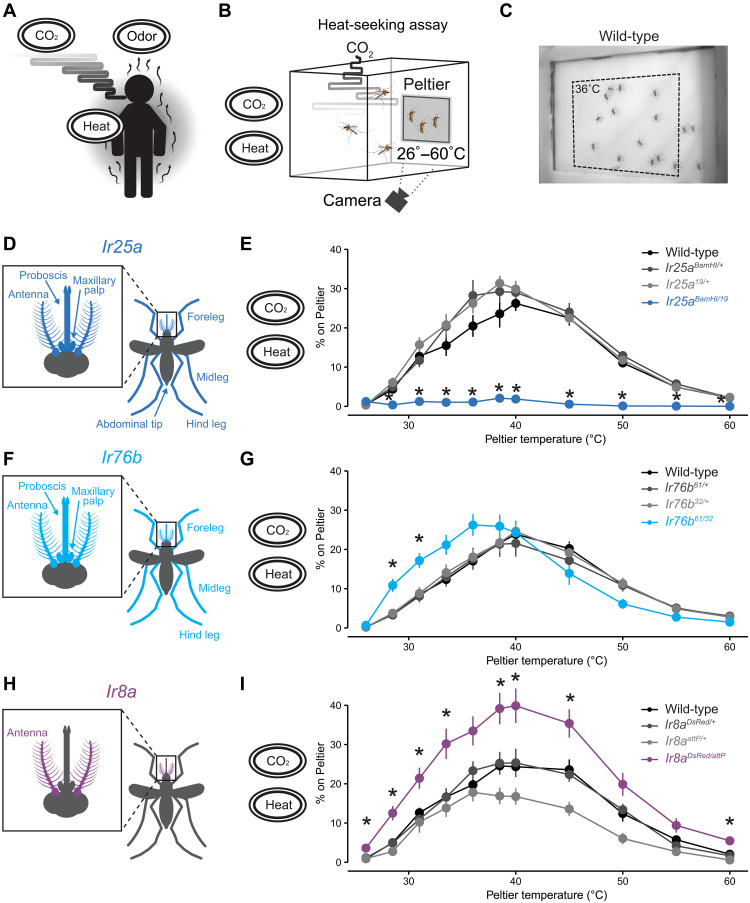
IRs tune thermotaxis behavior. (**A**) Female mosquitoes use redundant human-emitted cues (CO_2_, odor, and heat) to pursue blood meal. The black ovals with sensory cues displayed here and throughout the manuscript show the sensory stimuli present in the corresponding behavioral experiment. (**B**) Schematic of heat-seeking assay apparatus (30 cm^3^). (**C**) Representative experimental image of wild-type female mosquitoes on and near the Peltier (dotted line) set at 36°C. (**D**, **F**, and **H**) Schematic of female body parts that express *Ir25a* (D), *Ir76b* (F), and *Ir8a* (H). (**E**, **G**, and **I**) Percent of mosquitoes of indicated genotypes on Peltier during seconds 90 to 180 of stimuli of indicated temperature (mean ± SEM, *n* = 5 to 13 trials per genotype; data points marked with * indicate that the mutant differs significantly from all other tested genotypes within each tested temperature at *P* < 0.05; one-way analysis of variance (ANOVA) with Tukey’s HSD post hoc test). See also fig. S1 and movie S1.

Mosquitoes are sensory specialists that detect and integrate diverse host-emitted cues, most notably, human-emitted odors, carbon dioxide (CO_2_), and body heat (Fig. 1A). Unlike many other organisms, female mosquitoes need substantial blood meals to initiate egg production and can feed multiple times during their lifetime. This characteristic makes *Aedes aegypti* mosquitoes highly effective vectors for transmitting arboviruses such as dengue, yellow fever, Zika, and chikungunya, and makes *Anopheles gambiae* mosquitoes dangerous vectors of the *Plasmodium* malaria parasites. Mosquitoes rely on three large multigene families to detect human-emitted cues: gustatory receptors (GRs), odorant receptors (ORs), and ionotropic receptors (IRs). These proteins form multisubunit ligand-gated ion channel complexes featuring one or more ligand-selective subunits and an obligatory co-receptor subunit. While GRs are generally used for taste cues, members of this gene family form a heteromultimeric receptor that detects carbon dioxide (CO_2_) (*13*–*15*). In *Aedes aegypti*, there are 72 GRs, and the identity and role of co-receptors in this gene family are not well understood. In contrast, co-receptors for the OR and IR gene family are understood to be central to their function. Each insect species can have hundreds of ligand-selective ORs and IRs but only one OR co-receptor (Orco) and three IR co-receptors (IR8a, IR76b, and IR25a) (*16*). The co-receptor subunit is required for the assembly and trafficking of functional ion channel complexes to the plasma membrane, and mutations of Orco and individual IR co-receptor subunits in *Drosophila melanogaster* disrupt the assembly of functional receptors (*16*, *17*). In the case of *Aedes aegypti*, there are 116 ligand-selective ORs and 132 ligand-selective IRs (*18*). These large gene families of chemosensory receptors enable mosquitoes to a diversity of human-emitted cues. ORs generally respond to esters, alcohols, ketones, and aldehydes, while IRs have very flexible ligand tuning and have been shown to detect acids, amines, and physical stimuli, such as humidity and temperature (*19*). Because of the dependence of the functional receptor complex on the co-receptor, mutation of a single co-receptor gene can impair an insect’s ability to detect entire classes of human-emitted sensory cues.

With the advent of genome engineering in this nonmodel organism (*20*, *21*), it has been possible to show that genetic disruption of each sensory modality in isolation has modest effects on mosquito attraction to humans. Mosquitoes lacking the CO_2_ receptor GR3 continue to exhibit attraction to humans in laboratory attraction assays and an enclosed semifield assay designed to replicate a naturalistic human living environment (*13*). Mosquitoes with a loss-of-function mutation in *Orco* retain their attraction to humans but lose the strong preference for humans over nonhuman animals and are insensitive to the volatile effects of the insect repellent DEET (*N*,*N*-diethyl-meta-toluamide) (*20*). *Ir8a* mutants exhibit severe deficiencies in detecting lactic acid, a major component of human skin odor, but retain partial attraction to humans (*22*). *Ir76b* mutants remain attracted to humans despite losing their ability to feed on blood (*23*). *Ir21a* and *Ir93b* mutants experience reduced heat and humidity-seeking behavior but maintain their overall attraction to humans (*24*, *25*). The recent discovery of extensive coexpression of ORs and IRs in single sensory neurons may explain this functional redundancy (*26*, *27*). In addition, out of many sensory cues emitted by humans, the detection of just two cues is sufficient to initiate and synergize the drive for human-seeking behavior (*13*).

In this study, we found and investigated cross-modal sensory compensation between olfaction and thermosensation in the context of human-seeking behavior. We found that mutating each of the three IR co-receptors leads to different effects on thermotaxis behavior, ranging from complete loss of heat seeking (*Ir25a*), to a shift in preference to lower temperatures (*Ir76b*), or enhancement in sensitivity to heat (*Ir8a*). *Orco* mutant mosquitoes, which lack a functional OR pathway, show a remarkable increase in attraction to humans, which was fully attributable to their heightened sensitivity to heat. Contrary to our current understanding of insect thermosensation that points to the antenna as the major heat-sensitive organ, we found that mosquitoes use their forelegs as an essential sensory structure for heat detection in the range of human skin temperature. The heightened heat-seeking behavior in *Orco* mutants is accompanied by enhanced thermosensitivity in these foreleg neurons. Comparative gene expression studies between wild-type and *Orco* mutant legs showed that both *Ir25a* and the ligand-specific IR subunit *Ir140* were up-regulated in *Orco* mutant legs. Last, we determined that *Ir140* plays a substantial role in general heat-seeking behavior and that the enhanced thermosensitivity in *Orco* mutants is lost in *Ir140* mutants. Because *Orco* is only sparsely expressed in the leg, compensation is likely due to long-range communication between head appendages and the foreleg. This mechanism allows these dangerous vector insects to maintain their overall effectiveness in human-seeking behavior even when one sensory modality is compromised.

## RESULTS

### IRs tune mosquito thermotaxis behavior

Our work began with the aim to identify genes involved in mosquito thermal attraction at the specific thermal range of human skin temperature (34° to 37°C) (*28*) using a previously described assay that monitors mosquitoes landing on a warmed Peltier element in a cage supplemented with CO_2_ (*13*, *29*). This system can measure the attraction of mosquitoes to thermal stimuli by heating the Peltier element to a temperature ranging from ambient (26°C) to noxious (60°C) temperatures, and quantifying landing events (Fig. 1, B and C, and movie S1). Using this system, we took a candidate gene approach by measuring the thermotaxis behavior of mosquitoes carrying loss-of-function mutations in the three major IR co-receptors—*Ir25a*, *Ir76b*, and *Ir8a*.

First, we looked at a broadly expressed IR co-receptor subunit, *Ir25a* (Fig. 1D) (*30*). *Ir25a* is required for detecting acids, amines, humidity, cooling, and temperature-synchronized circadian clock oscillation in *Drosophila melanogaster* (*16*, *31*–*35*). *Ir25a* also mediates amine detection and human odor attraction in *Aedes aegypti* (*26*, *36*). The role of *Ir25a* in thermotaxis behavior in any mosquito species has not been examined. Using the heat-seeking assay, we found that *Ir25a* mutants failed to locate the heated Peltier element at all temperatures tested (Fig. 1E). *Ir25a* mutants display normal CO_2_-evoked flight activity and noxious heat detection, suggesting that the failure to respond to heat is not due to locomotor deficits or a lack of behavioral participation (fig. S1). These results indicate that *Ir25a* is required to respond to all physiologically relevant temperatures in the context of our heat-seeking assay.

We next looked at another broadly expressed IR co-receptor subunit, *Ir76b* (Fig. 1F). *Ir76b* is required for the detection of pH, amino acids, fatty acids, and salts in *Drosophila melanogaster* (*37*–*40*) and mediates amine detection and blood-feeding behavior in *Anopheles coluzzii* (*23*). The role of *Ir76b* in *Aedes aegypti* thermosensation has not been examined. *Ir76b* mutants showed normal responses to temperatures above 40°C but were more sensitive at lower temperatures (Fig. 1G). This effect was specific to lower temperatures between 28.5° and 31°C. These results suggest that *Ir76b* may be required to tune *Aedes aegypti* temperature preference toward human skin temperature.

Last, we looked at the antenna-enriched IR co-receptor subunit, *Ir8a*, which is required for lactic acid detection in *Drosophila melanogaster* and *Aedes aegypti* (*16*, *22*, *41*) (Fig. 1H). Unlike *Ir25a* and *Ir76b* mutants, *Ir8a* mutants displayed enhanced heat-seeking behavior broadly across most temperature ranges tested (Fig. 1I). Our analysis of heat-seeking behavior using our assay in these three IR co-receptor subunit mutants revealed distinct thermotaxis patterns for each mutant, implying that each IR co-receptor subunit contributes uniquely to the precise regulation of heat-seeking behavior in *Aedes aegypti*.

### *Orco* mutants display enhanced heat-seeking behavior

**Fig. 2.**
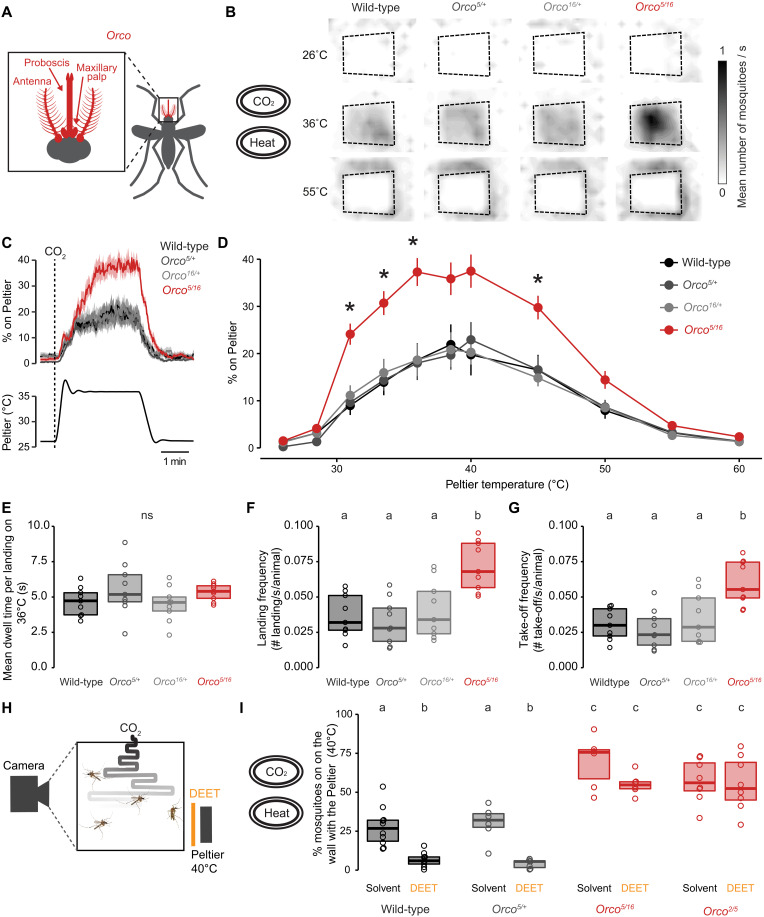
*Orco* mutant mosquitoes display enhanced heat-seeking behavior. (**A**) Schematic of female body parts that express *Orco*. (**B**) Heatmaps showing mean mosquito occupancy for the indicated genotypes on the Peltier (dotted lines) and surrounding area at indicated Peltier temperature during seconds 90 to 180 of each stimulus period. (**C**) Mean ± SEM percentage of mosquitoes of indicated genotypes on Peltier (top) during the 36°C trial (bottom). A 20-s pulse of CO_2_ was applied at the beginning of each stimulus period. (**D**) Percent of mosquitoes of indicated genotypes on Peltier during seconds 90 to 180 of stimuli of indicated temperature (mean ± SEM, *n* = 9 trials per genotype; data points marked with * indicate that the mutant differs significantly from all other tested genotypes within each tested temperature at *P* < 0.05; one-way ANOVA with Tukey’s HSD post hoc test). (**E** to **G**) Mean dwell time (E), landing frequency (F), and take-off frequency (G) of indicated genotypes on the Peltier surface during the 36°C trial (*n* = 9 trials per genotype). (**H**) Schematic representation of the modified heat-seeking assay (28 cm^3^) in the presence of DEET. (**I**) Percent of mosquitoes of indicated genotype and treatment groups heat-seeking during a 40°C trial (*n* = 6 to 10 trials per genotype). Data are plotted as scatter-box plots (individual data points, median as horizontal line, interquartile range as box) for (E) to (G) and (I). Data labeled with different letters differ significantly (*P* < 0.05; one-way ANOVA with Tukey’s HSD post hoc test). See also movie S2.

We next asked whether the Orco co-receptor is required for heat-seeking behavior in *Aedes aegypti*. In *Aedes aegypti*, *Orco* is mainly expressed in the antenna, proboscis, and maxillary palp (Fig. 2A) (*20*, *26*). *Orco* mutants displayed enhanced heat-seeking behavior compared to their genetic controls at 36°C (Fig. 2B and movie S2). This enhancement was sustained during the entire duration of the heat stimulus period (Fig. 2C). Furthermore, *Orco* mutants showed enhanced heat-seeking behavior, specifically toward human skin temperatures, and displayed normal avoidance behavior at the noxious temperature range (Fig. 2, B and D).

Next, we investigated which aspect of mosquito heat-seeking behavior was specifically altered in *Orco* mutants. We first tested whether *Orco* mutants spent longer on the heated surface by calculating the average dwell time per landing event for each animal at human skin temperature. Our analysis revealed that *Orco* mutants spent the same time as controls on the Peltier element per landing event (Fig. 2E). Another possibility was that *Orco* mosquitoes engaged in landing events more frequently than their genetic controls. We analyzed the final 60 s of each stimulus period and calculated both landing and take-off event frequencies for each animal. *Orco* mutants display increased landing and take-off frequencies, demonstrating that *Orco* mutants had persistent and sustained engagement toward the heated Peltier element throughout the heat trial (Fig. 2, F and G).

Given the marked increase in the attraction of *Orco* mutants to heat, we next asked whether the insect repellent DEET deters these mutants from heat-seeking behavior. Unlike wild-type animals, *Orco* mutants are attracted to human odor in the presence of DEET; however, whether volatile DEET interferes with heat seeking independently is not known (*20*). We designed a modified version of the heat-seeking assay by placing a source of DEET in front of the Peltier device set at 40°C (Fig. 2H). This source of DEET was placed across a spacer to avoid direct DEET contact by the mosquitoes. Contact chemorepellency is mediated by the mosquito leg and does not require *Orco* function (*42*). As expected, we found that DEET was sufficient to drive mosquitoes away from heat in wild-type and heterozygous controls. However, *Orco* mutant mosquitoes retained their enhanced heat-seeking behavior even in the presence of DEET (Fig. 2I). We replicated this result with another heteroallelic mutant, *Orco^2/5^* (Fig. 2I). These data indicate that enhanced heat seeking is unaffected by *Orco*-mediated repellency, thus depending on the absence of *Orco*. In addition, these data demonstrate the critical role of *Orco* in DEET detection and avoidance behavior in the presence of multimodal human-related cues (*20*).

### *Orco* mutants display enhanced human-seeking behavior

**Fig. 3.**
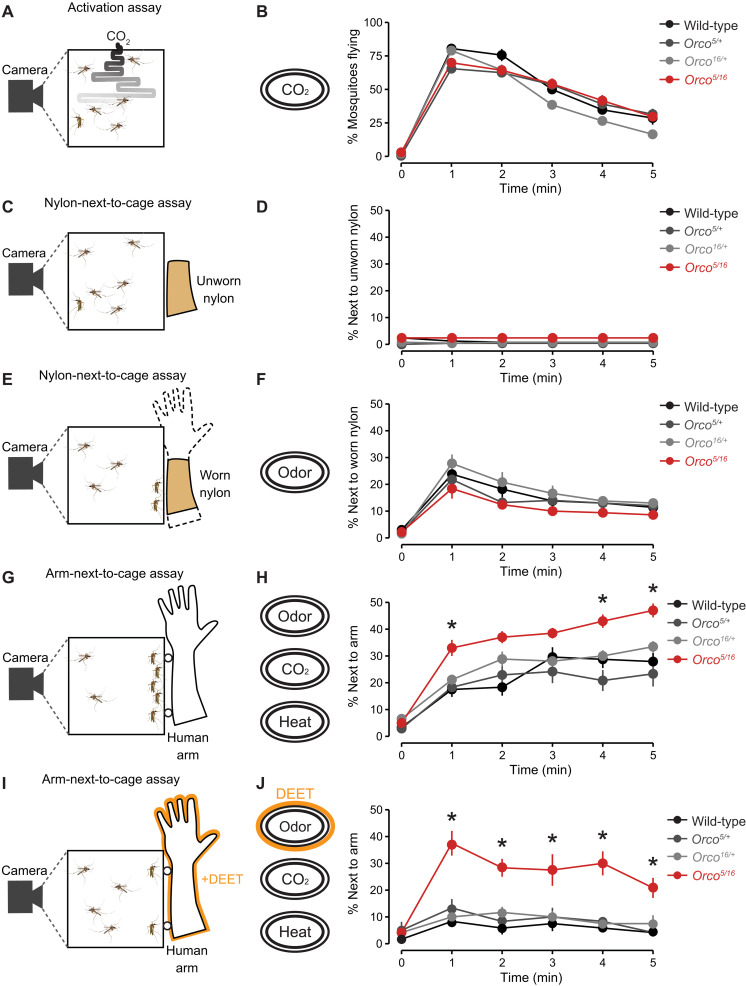
Human attraction is enhanced in *Orco* mutant mosquitoes. (**A**) Schematic of the CO_2_ activation assay. (**B**) Percent mosquitoes flying in the presence of CO_2_ quantified every minute (mean ± SEM, *n* = 10 trials per genotype). (**C** and **E**) Nylon-next-to-cage assay schematic with unworn (C) and worn (E) nylon. (**D** and **F**) Percent mosquitoes next to unworn [(D), *n* = 10 trials per genotype] or worn [(F), n = 5 trials per genotype] nylon, quantified once per minute (mean ± SEM). (**G** and **I**) Arm-next-to-cage assay schematic with human arm without (G) or with 10% DEET (I). (**H** and **J**) Percent mosquitoes next to the human arm without [(H), *n* = 10 to 13 trials per genotype] or with 10% DEET [(J), *n* = 6 trials per genotype] quantified once per minute (mean ± SEM). In (D), (F), (H), and (J), data points marked with * indicate that the mutant differs significantly from all other tested genotypes within each tested time point at *P* < 0.05; one-way ANOVA with Tukey’s HSD post hoc test). See also fig. S2.

We next investigated sensory cues of different modalities to test the specificity of the enhanced thermotaxis behavior displayed by *Orco* mutants. Using the CO_2_ activation assay, we tested the activity and arousal of *Orco* mutants in response to a brief 20-s pulse of CO_2_ (Fig. 3A and fig. S2A). CO_2_-evoked flight activity and total distance traveled in response to CO_2_ were indistinguishable from genetic controls (Fig. 3B and fig. S2, B and C). We further tested whether attraction toward human odor was altered in *Orco* mutants using the nylon-next-to-cage assay (Fig. 3, C and E). A previous study showed that *Orco* mutants show normal attraction to humans but are impaired in discriminating human from nonhuman odors (*20*). Consistent with these previous observations, *Orco* mutants show normal attraction toward human-worn nylon (Fig. 3, E and F). This attraction was specific to host odors because mosquitoes were not attracted to unworn nylon (Fig. 3, C and D). We further tested whether the enhanced thermosensitivity of *Orco* mutants enhances their ability to find live human hosts. To test this, we used an arm-next-to-cage assay with a human arm positioned close to the cage to allow mosquitoes to detect the human-emitted odor, CO_2,_ and body temperature without physical contact (Fig. 3G). We found that *Orco* mutants displayed enhanced human arm-seeking behavior compared to genetic controls (Fig. 3H). We further repeated the experiment in the presence of volatile DEET to test whether the enhanced human-seeking behavior is retained in the presence of this repellent (Fig. 3I). We found that *Orco* mutants were still capable of human arm-seeking behavior in the presence of DEET (Fig. 3J). These data, along with the heat-seeking data in the presence of DEET (Fig. 2I), suggest that enhancement of *Orco* human-seeking behavior is unaffected by DEET.

### Forelegs mediate thermotaxis behavior

**Fig. 4.**
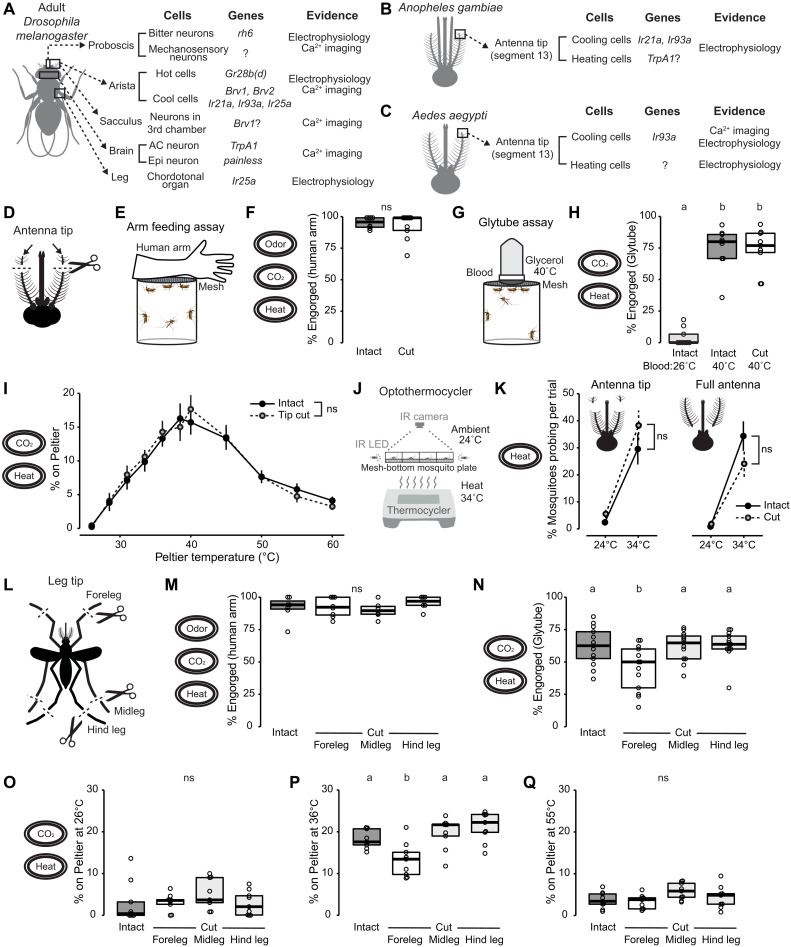
Forelegs but not antennae mediate *Aedes aegypti* heat-seeking behavior. (**A** to **C**) Sensory organs, genes, and experimental evidence for intrinsically thermosensitive neurons currently known in adult *Drosophila melanogaster* (*32*, *35*, *43*–*48*) (A), *Anopheles gambiae* (*24*, *25*, *52*) (B), and *Aedes aegypti* (*25*, *50*, *51*) (C). (**D**) Schematic of antennal tip removal, with distal three segments cut from both antennae. (**E**) Arm feeding assay schematic. (**F**) Percent engorged on the human arm of indicated antenna treatment (*n* = 8 to 9 trials per condition). (**G**) Glytube assay schematic. (**H**) Percent engorged on blood in the Glytube assay of indicated antenna treatment and blood temperature (*n* = 9 trials per condition). (**I**) Percent of mosquitoes of indicated antenna treatment on Peltier during seconds 90 to 180 of stimuli of indicated temperature (mean ± SEM, *n* = 8 to 9 trials per treatment; ns, unpaired *t* test at each tested temperature). (**J**) Schematic of the optothermocycler assay. (**K**) Percent probing in response to ambient temperature and heat at 34°C for antennal tip removal (left, *n* = 9 trials per condition) and full antenna removal (right, *n* = 8 trials per condition). Data are plotted as means ± SEM (ns, unpaired *t* test comparing cut and intact mosquitoes at 34°C). (**L**) Tarsal removal schematic, with distal three tarsal segments cut from each pair of legs. (**M**) Percent engorged on the human arm of indicated tarsal treatment (*n* = 6 to 8 trials per condition). (**N**) Percent engorged on blood in the Glytube assay of indicated tarsal treatment (*n* = 13 trials per condition). (**O** to **Q**) Percent of mosquitoes of indicated genotypes on Peltier during the 26°C (O), 36°C (P), or 55°C (Q) trials. *n* = 9 trials per genotype. Data are plotted as scatter-box plots (individual data points, median as horizontal line, interquartile range as box) for (F), (H), and (M) to (Q). Data labeled with different letters are significantly different (*P* < 0.05; one-way ANOVA with Tukey’s HSD post hoc test). See also fig. S3.

Previous studies in *Drosophila melanogaster* have identified intrinsically thermosensitive neurons activated by diverse thermoreceptors and ion channels (Fig. 4A) (*32*, *35*, *43*–*49*). In mosquitoes, prior studies identified a set of thermosensitive neurons at the most distal antennal segment that responded to cooling or heating air (Fig. 4, B and C) (*24*, *25*, *50*–*52*). Because *Orco* is expressed in the antenna, we hypothesized that loss of *Orco* may directly affect the function of thermosensitive neurons at the antennal tip. However, none of the previous studies investigated the direct roles and requirements of these thermosensitive neurons in the context of thermotaxis behaviors. To test this, we removed the three most distal antenna segments by cutting off the tip (Fig. 4D) and performed behavioral assays. We first wanted to confirm that antennal tip removal had minimal consequences on the overall behavior of the mosquitoes using an arm feeding assay where we measured the ability of the antennal tip-cut mosquitoes to blood-feed on a human arm (Fig. 4E). Antennal tip-cut mosquitoes engorged on human arms comparable to fully intact animals (Fig. 4F). This result confirms that antennal tip removal had minimal effect on overall human-seeking behavior, consistent with previous reports (*53*, *54*).

We then investigated the role of antennal tip neurons in thermotaxis behavior, first using a Glytube assay, an artificial blood-feeding system that provides mosquitoes with warm blood in the presence of CO_2_ (Fig. 4G) (*55*). Unexpectedly, we found that antennal tip-cut animals still engorged on blood at comparable levels to fully intact mock-treated animals (Fig. 4H).

We then turned to the heat-seeking assay to monitor heat-seeking behavior during each heat stimulus. Consistent with Glytube assay results, antenna-tip-cut mosquitoes displayed normal heat-seeking behavior, indistinguishable from fully intact mock-treated controls at all temperatures tested (Fig. 4I). Because our data show that the first three segments of the antennal tip are dispensable for arm-feeding, artificial blood-feeding and heat-seeking assays, we wanted to know whether any portion of the antenna was required to detect thermal information. Because complete antenna removal disables mosquitoes from flying (*53*, *54*), we turned to the opto-thermocycler assay (*56*). This assay tracks thermotaxis behavior using probing movement as a proxy for heat detection at high temporal and spatial resolution without requiring flying behavior (Fig. 4J). The antennal tip-cut animals displayed probing behavior indistinguishable from mock-treated controls, consistent with our other thermotaxis assays. Furthermore, animals with the entire antenna removed also detected and responded by displaying probing behavior to a thermal stimulus indistinguishable from fully intact mock-treated controls (Fig. 4K). These experiments suggest that the antennal tip and even the entire antenna are dispensable in *Aedes aegypti* heat detection using the optothermocycler assay.

We further tested the possibility that other sensory appendages are responsible for mosquito thermotaxis behavior. We focused on the legs because these sensory appendages make direct contact with heated surfaces, and a previous study showed that *Drosophila melanogaster* leg neurons respond directly to thermal stimuli (*32*). Ticks also have a unique sensory organ on their distal leg segment called Haller’s organ that functions as a heat sensor (*57*). To determine whether legs contribute to thermotaxis behavior in *Aedes aegypti*, we removed the three most distal segments from each pair of legs – forelegs, midlegs, or hind legs (Fig. 4L). Cutting the tips of a single pair of legs had no significant effect on human-seeking and blood-feeding behavior on a live human arm (Fig. 4M). However, animals with foreleg tips cut had a significant reduction in their ability to engorge on blood in the Glytube assay, where the only cues presented to these animals were heat and CO_2_ (Fig. 4N). Furthermore, animals with foreleg tips cut had a significant reduction in heat-seeking behavior to the Peltier element set to 36°C (Fig. 4P) but not at ambient (Fig. 4O) or at a noxious temperature (Fig. 4Q). Reduced heat-seeking behavior at 36°C was due to decreased landing and take-off frequencies, while the dwell time per landing event was not affected (fig. S3). These data suggest that mosquito forelegs contribute strongly to *Aedes aegypti* thermotaxis behavior.

### The neuroanatomy of the *Aedes aegypti* leg is uniquely organized

**Fig. 5.**
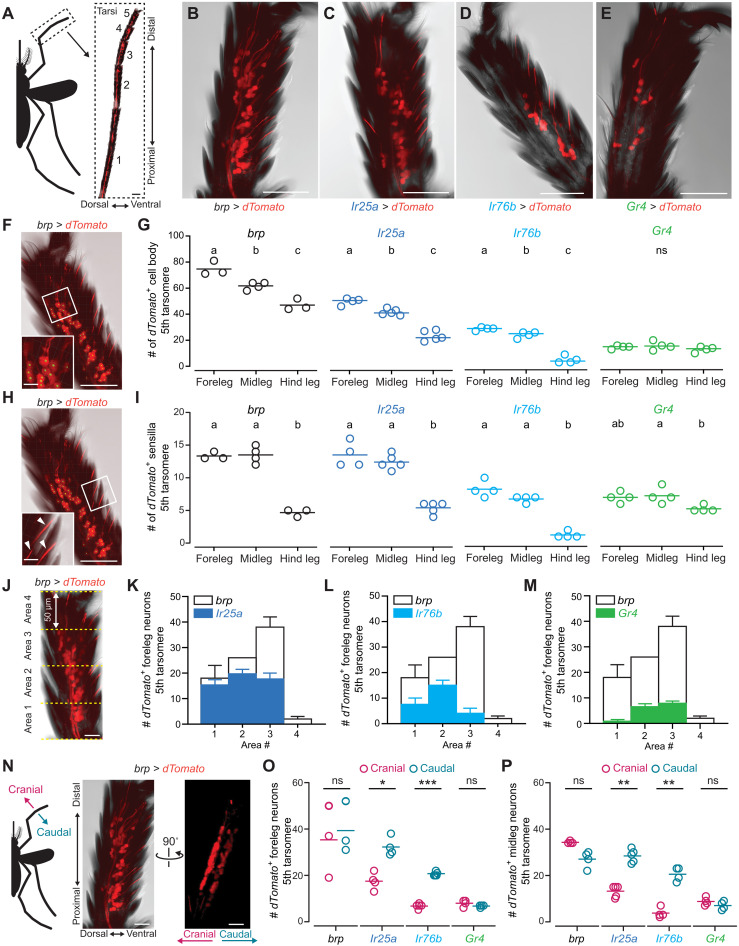
Morphology of tarsal neurons. (**A**) Tiled confocal image of tarsal segments from *brp*-*QF2w > QUAS-dTomato* mosquitoes expressing dTomato. Dorsal is left and distal is top with individual tarsomeres numbered from proximal to distal segments for all images. (**B** to **E**) Maximum-intensity projections of dTomato expression in the fifth tarsomere of forelegs in *brp*-*QF2w > QUAS-dTomato* (B), *Ir25a-QF2 > QUAS-dTomato* (C), *Ir76b-QF2 > QUAS-dTomato* (D), and *Gr4-QF2 > QUAS-dTomato* (E) animals. (**F** and **H**) Representative image of the fifth tarsomere used for cell body (F) and sensilla (H) quantification. (Inset) Magnification of area outlined in white. Green dots indicate cell bodies while arrowheads indicate individual sensilla. (**G** and **I**) Quantification of dTomato-labeled cell bodies (G) and sensilla (I) in the fifth tarsomere (*n* = 3 to 5 tarsi). Scatter plots indicate individual data points with mean as horizontal line. Statistical significance between groups are indicated by letters (*P* < 0.05; one-way ANOVA with Tukey’s HSD post hoc test). (**J** to **M**) Distribution of dTomato-expressing neurons along the proximal-distal axis of the fifth tarsomere in forelegs from *Ir25a-QF2 > QUAS-dTomato* (K), *Ir76b-QF2 > QUAS-dTomato* (L), and *Gr4-QF2 > QUAS-dTomato* (M). The fifth tarsomere is divided into four 50-μm areas indicated with yellow dotted lines (J). Bars outlined in black indicate *brp-*positive neurons in the same area from a different sample (*n* = 2 to 4 tarsi). (**N**) Schematic representation of the cranial-caudal axis and 3D projection of the fifth tarsomere shown in (B) rotated ~90°. (**O** and **P**) Number of dTomato-labeled cell bodies in individual planes in the fifth tarsomere of forelegs (O) and midlegs (P) labeled by the indicated driver lines (*n* = 3 to 5 tarsi). Scatter plots display individual data points with mean as a horizontal line (paired *t* test, *P < 0.05, **P < 0.01, and ***P < 0.005). Scale bars, 100 μm (A), 50 μm [(B) to (F), (H), (J), and (N)], and 10 μm [insets in (F) and (H)]. See also movie S3.

The neuroanatomy of *Aedes aegypti* legs has not been extensively studied. Insect legs comprise multiple segments—coxa, trochanter, femur, tibia, and tarsus. We focused on the tarsus, explicitly looking at the fifth tarsomere (Fig. 5A), which is the most distal tarsal segment and considered the primary site for chemoreception in the leg (*58*, *59*). We characterized the neuronal anatomy of the fifth tarsomere using a panel of chemosensory receptor driver lines, each expressing a fluorescent reporter, *dTomato*, in its corresponding neural population (Fig. 5, B to E). Specifically, we used *brp-QF2w > QUAS-dTomato* to label all neurons (*60*), *Ir25a-QF2 > QUAS-dTomato* and *Ir76b-QF2 > QUAS-dTomato* to label putative thermosensitive and chemosensitive cells (*26*), and *Gr4-QF2 > QUAS-dTomato* to label sugar-sensing neurons previously shown to induce appetitive behavior once activated (*61*). *Aedes aegypti* tarsi are densely covered with scales, and the neuronal cell bodies extend their dendrites into sensilla that decorate the leg.

Using the *brp-QF2w > QUAS-dTomato* reporter line, we quantified the number of neurons within the fifth tarsomere of each leg and observed a decreasing order of neuron count from forelegs to midlegs to hind legs (Fig. 5, F and G). We identified an average of 75 total neurons in the fifth tarsomere of the foreleg, 62 in the fifth tarsomere of the midleg, and 47 in the fifth tarsomere of the hind leg (Fig. 5, B and G). We then examined reporter expression for *Ir25a*, *Ir76b*, and *Gr4*. *Ir25a-*expressing neurons were found in roughly two-thirds of the total fifth tarsal neurons, with a sequential decrease from foreleg to midleg to hind leg (Fig. 5, C and G). *Ir76b* expression was found in one-third of the neurons of the foreleg and midleg and in fewer neurons in the hind leg (Fig. 5, D and G). In contrast, expression of the *Gr4* sugar receptor was equivalent in all three legs (Fig. 5, E and G).

In our images, we observed and quantified dendritic processes from cell bodies into their respective sensilla (Fig. 5H). As with the total neural count, the total sensilla count (*brp-*positive) was much higher in the fifth tarsomere of foreleg and midleg compared to hind leg (Fig. 5, B and I). The number of *Ir25a*-positive sensilla count mirrored that of *brp*-positive sensilla count, suggesting that *Ir25a*-positive neurons innervate all or most chemosensory sensilla (Fig. 5, C and I). *Ir76b*-positive sensilla represented half of the total sensilla counts in all three legs (Fig. 5, D and I). The *Gr4*-positive sensilla count represented half of the total *brp*-positive sensilla counts in the foreleg and midleg and had roughly the same number of hind leg sensilla (Fig. 5, E and I) (*26*).

We further investigated the spatial distribution of each chemoreceptor-expressing neuron within the fifth tarsomere. We divided the fifth tarsomere into 50-μm regions and counted the neurons in each region (Fig. 5J). *Ir25a*-expressing neurons were broadly distributed across the entire fifth tarsomere, while *Ir76b*-expressing neurons were more concentrated in the proximal tarsal region. *Gr4*-expressing neurons were primarily located in the distal area of the fifth tarsomere (Fig. 5, K to M). We noticed that the neurons were organized in two planes on each side of the tarsus (Fig. 5N and movie S3). To our knowledge, this has not been described in other insects. When the mosquito leg is outstretched, one side of the tarsus faces toward the head, and the other is oriented toward the caudal end of the abdomen. Thus, we named these two neural planes the “cranial” and “caudal” sides of the tarsus and quantified the number of labeled neurons per plane for each reporter line. While the overall number of foreleg neurons of the fifth tarsomere in each plane was similar, *Ir25a* and *Ir76b* represented a higher proportion of neurons on the caudal side compared to the cranial side, while *Gr4*-expressing neurons displayed an even distribution between the two sides (Fig. 5O). This cranial-caudal distribution was similar in the midleg (Fig. 5P).

### Mosquito leg neurons respond to heat

**Fig. 6.**
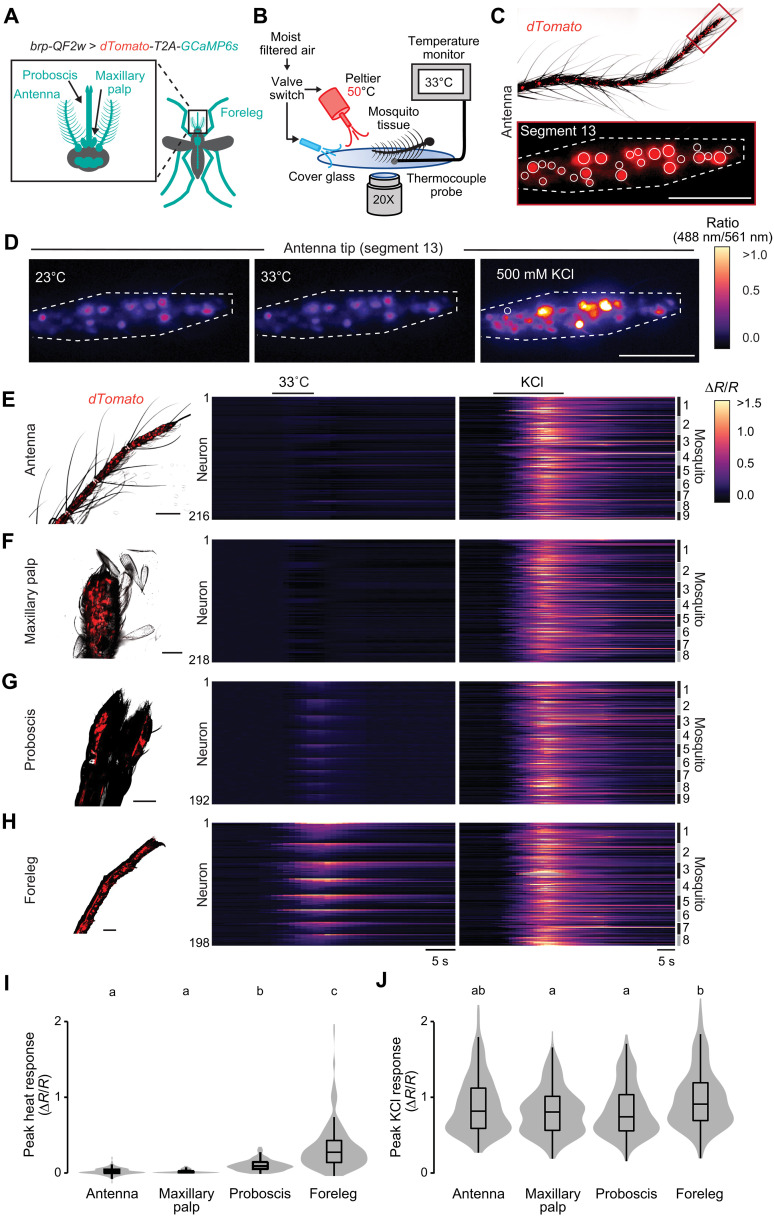
Tarsal neurons in the mosquito leg respond to heat. (**A**) Schematic of female body parts that express GCaMP6s in the pan-neuronal imaging strain *brp-QF2w > dTomato-T2A-GCaMP6s*. (**B**) Schematic of the ex vivo ratiometric sensory appendage Ca^2+^ imaging setup. (**C**) Representative image of a mosquito antenna (top) and the distal segment (bottom, segment 13) from a *brp-QF2w > dTomato-T2A-GCaMP6s* mosquito. Example regions of interest (ROIs) used to highlight individual neuron boundaries are indicated with solid lines, while the distal antenna segment is outlined with dotted lines. (**D**) Wide-field images of the antenna distal segment [from (C)] before, during heat stimulus, and after 500 mM KCl. Neuronal responses are shown as pseudo-colored images, with antenna distal segment outlined by dotted lines. (**E** to **H**) Wide-field dTomato image (left) and heatmap showing the calcium imaging response for individual neurons over time to heat (middle) and 500 mM KCl (right) in the antenna distal segment [(E), *n* = 216 neurons, nine antennae from nine mosquitoes], maxillary palp [(F), *n* = 218 neurons, eight maxillary palps from eight mosquitoes], proboscis [(G), *n* = 192 neurons, nine proboscises from nine mosquitoes], and forelegs [(H), *n* = 198 neurons, eight forelegs from eight mosquitoes]. Neuronal responses are clustered by individual appendages from each mosquito, then ordered from highest to lowest heat responding neurons, maintaining neuron identity between heat and KCl responses. (**I** and **J**) Peak heat (I) and 500 mM KCl (J) responses from the indicated sensory appendages. Data are presented as violin-box plots (median as horizontal line, interquartile range as box, and 5th and 95th percentiles as whiskers). Data labeled with different letters are significantly different (*P* < 0.05, Kruskal-Wallis test with Dunn’s multiple comparisons). Scale bars, 50 μm for (C) to (H). See also fig. S4 and movie S4.

We next asked which neurons in each of the main *Aedes aegypti* sensory appendages responded to heat. We used the pan-neuronal imaging line *brp-QF2w > QUAS-dTomato-T2A-GCaMP6s* to measure Ca^2+^ response as a proxy for neuronal activity in response to heated air in the four main sensory appendages—antenna, maxillary palp, proboscis, and legs (Fig. 6A). We developed a functional imaging setup that allowed us to acquire GCaMP and dTomato fluorescence signals while applying heated air over the samples (Fig. 6B). We report the change in the ratio of the GCaMP signal over the dTomato signal (*R = GCaMP6s/*dTomato) for individual neurons, normalized to baseline values (Δ*R/R*) to correct for movement artifact and temperature-evoked intrinsic changes in fluorescence signals (Fig. 6C and fig. S4, A to C). This approach has previously been used to study thermosensitivity in *C. elegans* thermosensory neurons (*62*, *63*).

We first tested whether antenna tip (segment 13) neurons responded to heated air. Unlike previous studies, which reported neuronal activity in response to heat using electrophysiological techniques (*50*, *51*), we did not detect robust calcium activity in response to warm air using wide-field calcium imaging. However, the antenna samples showed strong responses when activated with 500 mM KCl, indicating the viability of the samples in this imaging setup (Fig. 6, D and E). We then tested the potential for heat-evoked neuronal responses in other sensory structures. We did not detect calcium activity in the maxillary palp (Fig. 6F), while the proboscis showed weak responses to heated air (Fig. 6G). Among all sensory appendages tested, and consistent with our behavioral data, foreleg neurons across all three distal segments responded robustly to heated air (Fig. 6H) and displayed reproducible responses in each trial (fig. S4D). This response was independent of any mechanical artifact from valve switching between two air outlets, as the neuronal response returned once the heat source was turned back on (fig. S4, E and F). Of the tested sensory appendages, foreleg neurons displayed the strongest heat-evoked activity (Fig. 6I), and all four sensory appendages showed activation in response to 500 mM KCl, confirming neuronal viability (Fig. 6J). These functional imaging data further suggest a critical role for foreleg neurons during thermotaxis behavior.

**Fig. 7.**
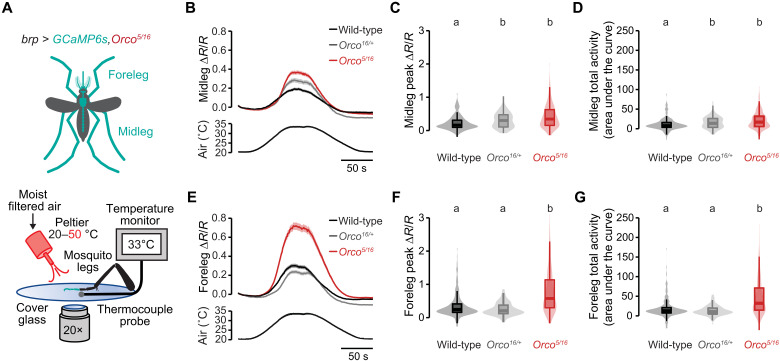
*Orco* mediates enhanced thermosensitivity in the mosquito foreleg. (**A**) Schematic of female body parts expressing GCaMP6s in the pan-neuronal imaging strain *brp-QF2w > dTomato-T2A-GCaMP6s* in the *Orco^5/16^* mutant background (top) and a schematic of the ex vivo ratiometric sensory appendage Ca^2+^ imaging setup (bottom). (**B** to **D** and **E** to **G**) *Orco* mutant midleg [(B) to (D)] and foreleg [(E) to (G)] responses to warm air showing average Ca^2+^ responses over time [(B), midleg; (E), foreleg], peak responses [(C), midleg; (F), foreleg], and total activity [(D), midleg; (G), foreleg] of the indicated genotypes [(B) to (D): *n* = 91 to 265 total ROI from 4 to 10 midlegs from each genotype, one midleg taken from each mosquito; (E) to (G): *n* = 102 to 260 total ROI from 4 to 10 forelegs from each genotype, one foreleg taken from each mosquito]. Data are plotted as means ± SEM for (B) and (E) and as violin-box plots (median as horizontal line, interquartile range as the box, and 5th and 95th percentiles indicated as whiskers) for (C), (D), (F), and (G). Data labeled with different letters are significantly different (*P* < 0.05, Kruskal-Wallis test with Dunn’s multiple comparisons).

We then asked whether the foreleg exhibits enhanced thermosensitivity in *Orco* mutants. To test this, we generated heteroallelic *Orco* mutant pan-neuronal imaging mosquitoes by crossing two different *Orco* mutant alleles into the *brp-QF2w > QUAS-dTomato-T2A-GCaMP6s* imaging background strain (Fig. 7A). We modified the imaging setup to apply a slow heat ramp using a single Peltier outlet to capture any differences in heat-evoked activation thresholds in *Orco* mutant leg neurons.

Using these *Orco* mutant pan-neuronal GCaMP mosquitoes and the modified imaging setup, we found that both heterozygous and *Orco* mutant midlegs, although indistinguishable from each other, displayed slightly higher peak response and total activity in response to heated air than wild-type controls (Fig. 7, B to D). This heterozygous effect was unexpected, as *Orco* typically displays a fully recessive phenotype (*20*), but the effect size in the midlegs was small for both homozygous and heterozygous mutants. In contrast, peak amplitude and total activity were strongly enhanced in *Orco* mutant forelegs compared to both wild-type and heterozygous controls (Fig. 7, E to G). These data suggest that the enhanced thermosensitivity in foreleg neurons contributes to the increased thermotaxis behavior observed in *Orco* mutants.

### Ionotropic receptor genes are up-regulated in the *Orco* mutant legs

**Fig. 8.**
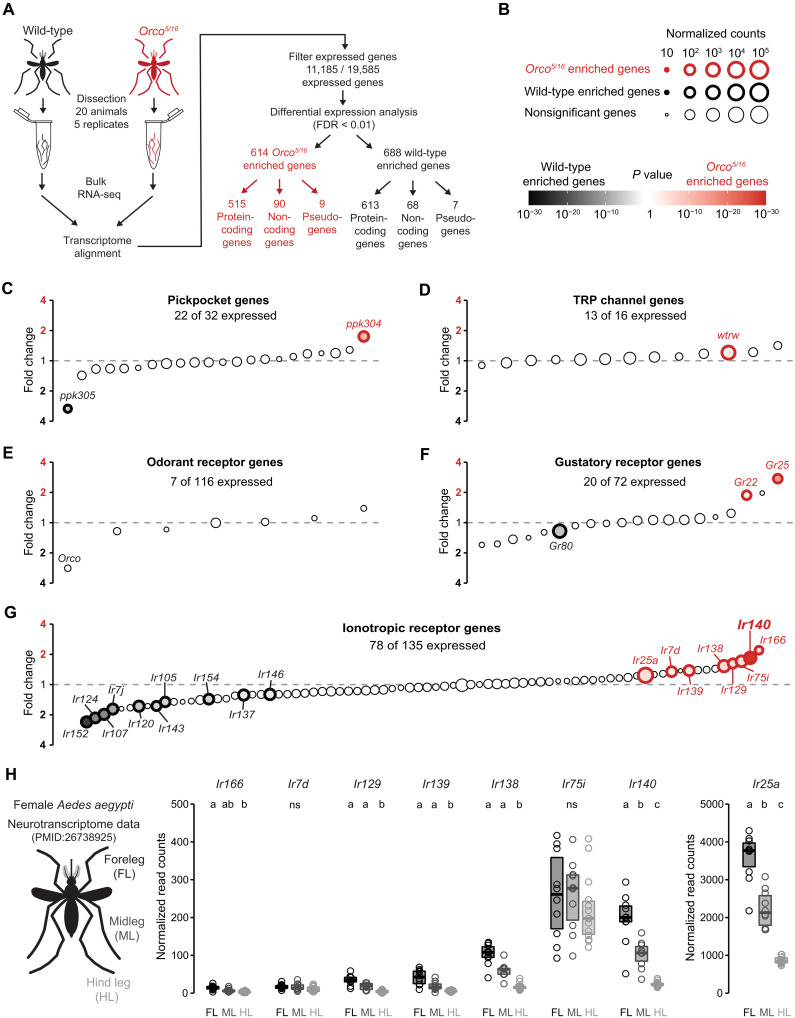
*Aedes aegypti* chemosensory receptor expression changes in the *Orco* mutant legs. (**A**) Workflow of pooled foreleg and midleg bulk RNA-seq comparing gene expression between wild-type and *Orco^5/16^* mutant mosquito tarsi. (**B**) Legend describing bulk RNA-seq data presented in (C) to (G). Normalized read counts are presented as size, differentially expressed genes are outlined in bold colors, and the degree of differential expression is represented with a color gradient. Read counts were normalized using the DESeq2 median of ratio method. (**C** to **G**) Gene expression comparisons between wild-type and *Orco^5/16^* mutant mosquito tarsi for Pickpocket genes (C), TRP channels (D), ORs (E), GRs (F), and IRs (G). Genes are listed from left to right by increasing fold-change enrichment in *Orco^5/16^* tarsi compared to wild type. The *y* axis is labeled with actual fold change values on a log_2_ scale. (**H**) *Orco^5/16^–*up-regulated IR gene expression in the indicated tissues from the female leg neurotranscriptome dataset (*30*). Data labeled with different letters are significantly different (*n* = 10 to 14 replicates per leg, FDR < 0.01, pairwise DESeq2 comparison). See also figs. S5 and S6.

We next investigated the potential molecular mechanisms underlying the enhanced neuronal activity in *Orco* mutant foreleg neurons. We performed bulk RNA sequencing (RNA-seq) from pooled whole forelegs and midlegs isolated from wild-type and *Orco* mutant mosquitoes (Fig. 8A). Using a false discovery rate (FDR) cutoff of 0.01, we looked for differentially expressed genes, their expression level, and the degree of differential expression (Fig. 8B) and found 614 up-regulated and 688 down-regulated genes in *Orco* mutants (Fig. 8A).

We narrowed our search by focusing on known sensory receptor genes. Of the 32 *pickpocket* genes, 22 were detected, with *ppk304* up-regulated and *ppk305* down-regulated in *Orco* mutant legs (Fig. 8C). For transient receptor potential (TRP) channels, 13 of 16 genes were detected, with only *water witch* (*wtrw*) up-regulated in *Orco* mutant legs (Fig. 8D). ORs were mostly absent in the legs, with only 7 of 116 detected, and none were differentially expressed in *Orco* mutant legs (Fig. 8E). Trace levels of *Orco* transcripts were detected in our *Orco^5/16^* samples, but at a lower level compared to wild-type controls with the expected 5- and 16-bp deletion in mapped reads at each mutant allele locus, likely reflecting transcripts undergoing nonsense-mediated decay (fig. S5A). At the gene-wide comparison level, *Orco* did not meet our stringent FDR < 0.01 cutoff threshold but still displayed a strong trend toward down-regulation in the *Orco^5/16^* samples with adjusted *P* value at 0.028 and FDR < 0.05 (fig. S5B). Of the 72 GRs, only 20 were expressed, with *Gr22* and *Gr25* up-regulated and *Gr80* down-regulated in *Orco* mutant legs (Fig. 8F). In contrast, 78 of 135 IRs were expressed, with 8 up-regulated and 10 down-regulated in *Orco* mutant legs (Fig. 8G). These transcriptome data suggest that in the legs, IRs are under stronger regulation by the *Orco* mutation than other chemosensory receptor classes.

To further identify potential genes underlying the enhanced heat-evoked neuronal activity in *Orco* mutant forelegs, we reanalyzed the previously published *Aedes aegypti* neurotranscriptome dataset to quantify which *Orco–*up-regulated IR genes were also enriched in the forelegs. From this reanalysis, only *Ir25a* and *Ir140* were found to be enriched in the foreleg (Fig. 8H). This enrichment was specific to female samples as the male transcriptome dataset detected both *Ir25a* and *Ir140*, but the foreleg enrichment was less profound as the female samples (fig. S5C). The *Ir25a* expression data align with our *Ir25a* heat-seeking behavioral data, suggesting that *Ir25a* is a core co-receptor subunit essential for thermotaxis behavior (Fig. 1E).

### *Ir140* mediates heat-seeking behavior and enhanced *Orco* thermosensation

**Fig. 9.**
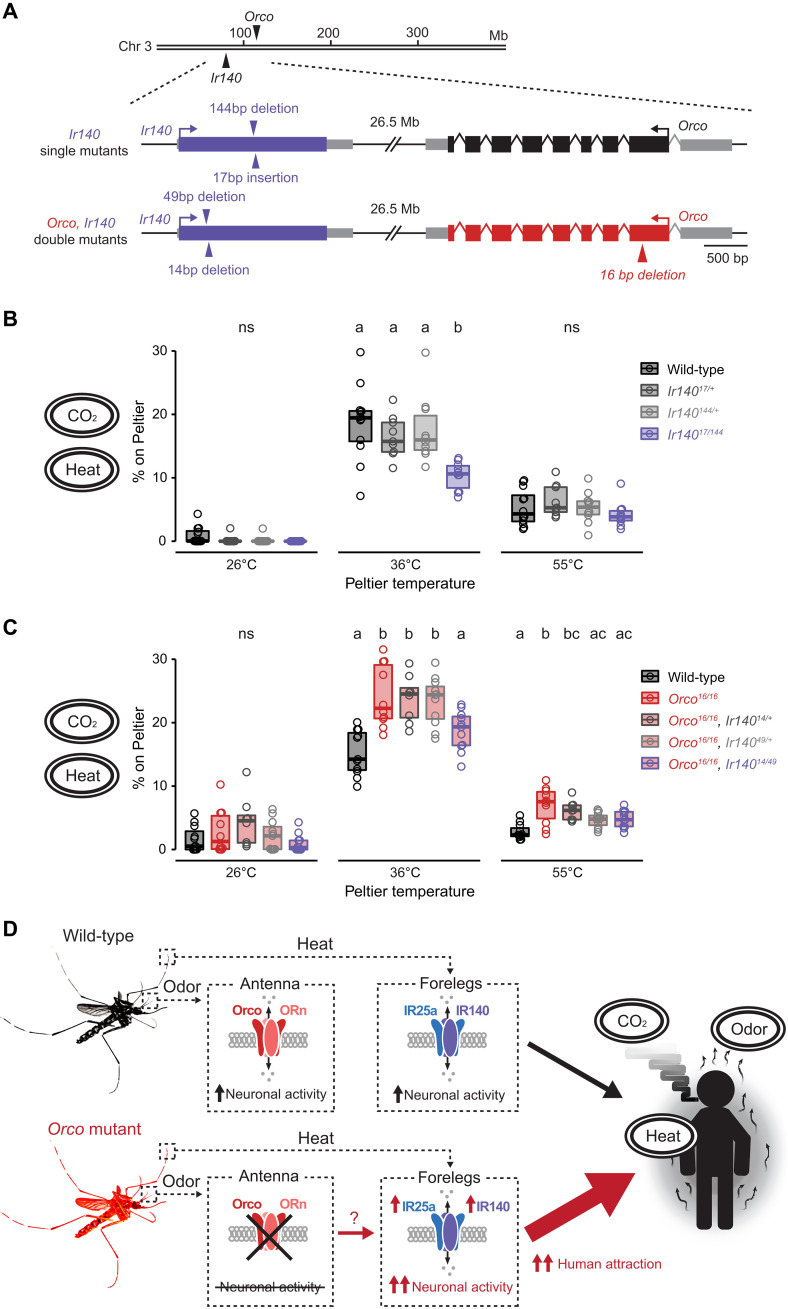
*Ir140* contributes to heat-seeking behavior and is required for the enhanced *Orco* mutant heat-seeking behavior. (**A**) Schematic of *Aedes aegypti Ir140* and *Orco* loci. Arrows indicate *Ir140* mutant alleles in wild-type and *Orco* mutant backgrounds. Introns are not drawn to scale. (**B** and **C**) Percent of mosquitoes of indicated genotype on Peltier during seconds 90 to 180 of stimuli of indicated temperature for single *Ir140* mutants [(B), *n* = 9 to 12 trials per genotype] and *Orco*, *Ir140* double mutants [(C), *n* = 8 to 11 trials per genotype]. (**D**) Summary and model of *Aedes aegypti* heat detection and enhanced thermosensitivity in *Orco* mutant mosquitoes. Data are plotted as scatter-box plots (individual data points, median as horizontal line, interquartile range as box) for (B) and (C). Data labeled with different letters are significantly different (*P* < 0.05; one-way ANOVA with Tukey’s HSD post hoc test at each tested temperature). See also figs. S7 and S8.

To test the role of *Ir140* in thermosensation, we used an enhanced CRISPR-Cas9 genome editing method that builds on our original method (*21*) to generate *Ir140* mutant mosquitoes that lack the functional Ir140 ligand-specific IR subunit. We isolated two *Ir140* mutant alleles, *Ir140^144^* and *Ir140^17^*, and used the heat-seeking assay to assess the thermotaxis behavior of heterozygous animals and heteroallelic *Ir140^17/144^* null mutants (Fig. 9A and fig. S7, A to C). *Ir140* mutants displayed a significant reduction in heat-seeking behavior compared to their genetic controls only when the Peltier was set at 36°C (Fig. 9B). This reduction in *Ir140* mutant heat-seeking behavior was attributed to decreased landing and take-off frequencies, with dwell time per landing event unaffected (fig. S8, A to C). These data suggest that *Ir140* is a key molecular player in *Aedes aegypti* thermotaxis behavior at human skin temperatures.

We further tested whether *Ir140* up-regulation could underlie the enhanced thermosensitivity observed in *Orco* mutants. Because *Orco* and *Ir140* are closely linked on the third chromosome, it was not feasible to simply cross *Ir140* mutant alleles into the *Orco* mutant background because of the low likelihood of recovering recombination events between these loci. Therefore, we generated *Orco*, *Ir140* double mutants by targeting *Ir140* using CRISPR-Cas9 genome editing in the *Orco^16^* mutant background strain (Fig. 9A and fig. S7, D to F). We isolated two *Orco, Ir140* double-mutant alleles, *Orco^16^, Ir140^14^* and *Orco^16^, Ir140^49^*, and tested the thermotaxis behavior of heteroallelic *Orco^16/16^, Ir140^14/49^* double mutants using the heat-seeking assay. While *Orco^16/16^*, and the heterozygous mutants displayed enhanced heat-seeking behavior, heteroallelic *Orco^16/16^, Ir140^14/49^* double-mutant animals failed to show this enhancement and were indistinguishable from wild-type controls (Fig. 9C and fig. S8, D to F). These results indicate that *Ir140* is a key molecular player involved in general thermosensitivity and is required for the enhanced thermosensitivity displayed by *Orco* mutant mosquitoes.

## DISCUSSION

### Cross-modal sensory compensation enhances mosquito attraction to humans

In this study, we establish an unexpected olfactory-to-thermal sensory compensation mechanism in *Aedes aegypti* mosquitoes. Our findings demonstrate that *Orco* mutant mosquitoes exhibit heightened thermosensitivity due to increased neuronal activity in their legs. In addition, we show that many IRs were differentially expressed in *Orco* mutant legs. Last, our study reveals the importance of *Ir140*, a ligand-specific IR subunit, in normal heat-seeking behavior and its necessity for the heightened thermosensitivity observed in *Orco* mutants (Fig. 9D). This cross-modal sensory compensation expands the behavioral strategies that *Aedes aegypti* can use in their persistent and seemingly unbreakable drive to seek humans. These observations align with prior work showing that disruptions in specific sensory receptors caused reductions in mosquito behavior related to the affected sensory modality but did not eliminate their overall attraction to humans (*13*, *24*, *25*, *36*). Coexpression of multiple chemosensory receptor gene families within a single olfactory sensory neuron may enable mosquitoes to rely on at least one chemoreceptor gene family if others become compromised (*26*, *27*). While it remains unknown whether receptor coexpression occurs in sensory neurons in the leg, the recent development of single-cell RNA-seq techniques for mosquito sensory neurons (*26*) may help to illuminate this.

### Is temperature detection between *Drosophila* and mosquitoes conserved?

Thermosensation has been extensively studied in *Drosophila melanogaster*, uncovering various genes and sensory organs involved in detecting environmental temperatures (Fig. 4A). TRP channels, an evolutionarily conserved class of ion channels, play diverse roles in thermosensation, ranging from noxious cold to heat sensation. For instance, *Painless* is essential for detecting noxious heat in both larvae and adults (*64*, *65*), while *TrpA1* is required for warmth detection via anterior cell (AC) neurons in the brain and for noxious heat avoidance behavior. (*45*, *47*, *66*–*68*). *Pyrexia* is necessary for noxious heat–evoked paralysis behavior (*69*). Cool detection through TRP channels is mediated by *Iav* in the chordotonal organ (*70*), with cool avoidance deficiencies observed in *Trp* and *Trpl* mutants (*71*), as well as *brv1*, *brv2*, and *brv3* mutants in cold cells within the arista (*46*). In addition, *Gr28b(d)* is involved in heat avoidance in adults (*44*, *45*), and opsins have been implicated in larval temperature preference and feeding behavior modulation (*43*, *72*, *73*). Another important class of ion channels, the IRs play roles beyond chemoreception of acids and amines and are involved in sensing physical stimuli such as humidity and temperature (*32*–*35*, *74*, *75*). For example, innocuous cool temperature detection and avoidance rely on *Ir21a*, *Ir93a*, and *Ir25a* in larval dorsal organ cool cells and adult arista cooling cells (*32*, *33*, *74*). Although it remains unclear whether IRs form intrinsically thermosensitive ion channels, a recent study reported that *Ir21a*, *Ir93a*, and *Ir25a* play crucial roles in neuronal morphogenesis (*35*). These IR subunits are required to form the specialized membrane contacts characteristic of cooling cell sensory endings in the arista, which underlie cooling-mediated neuronal excitability and thermal avoidance behavior.

Building on these studies in *Drosophila melanogaster*, recent studies have explored potential functional conservation of these genes in mosquitoes. In *Anopheles gambiae*, *Ir21a* and *Ir93b* are expressed in sensory neurons at the antennal tip, and mutations in these genes reduce neuronal activity in response to cooling air, leading to deficits in heat-seeking behavior at 37°C (*24*, *25*). In *Aedes aegypti*, *Ir93a*-expressing neurons in the antenna are thermosensitive, though the role of this ligand-selective IR in heat-seeking behavior remains untested (*25*). Furthermore, it is not known whether the ciliary morphology of antennal tip thermosensory neurons is altered in these *Ir21a* and *Ir93a* mosquito mutants, as in *Drosophila melanogaster*. In contrast to cooling detection, functional similarities in heat detection between *Drosophila melanogaster* and mosquito heat detection for human skin temperature is not well conserved. Unlike *Drosophila melanogaster TrpA1* mutants, *Aedes aegypti TrpA1* mutants exhibit normal thermotaxis behavior within the human skin temperature range (26° to 40°C) but fail to avoid temperatures over 40°C, indicating a partially conserved role for *TrpA1* as a noxious heat sensor in mosquitoes (*29*). In addition, *Gr19*, the *Aedes aegypti* homolog of the *Drosophila melanogaster Gr28b(d)* heat-avoidance gene, is not required for mosquito thermotaxis (*29*). These findings suggest that mosquito thermosensory mechanisms differ from those in flies, reflecting species-specific adaptations as mosquitoes are drawn to the warmth of human skin, while *Drosophila* strongly avoids this temperature range. Our current work identifies *Ir140* as a mosquito-specific gene mediating thermotaxis in *Aedes aegypti*. Given the complete abolishment of heat-seeking behavior in *Ir25a* mutants, we speculate that additional ligand-specific IR subunits may also play key roles in mosquito heat detection.

### What is the role of the antenna during thermotaxis behavior?

The mosquito antenna has long been considered the essential sensory appendage for mosquito thermotaxis. Earlier studies identified a pair of sensory neurons within the small coeloconic sensilla at the antennal tip that respond to air temperature changes (*24*, *50*–*52*). These studies revealed that one of these sensory neurons responds to rising air temperatures, while the other is sensitive to cooling (*50*–*52*). However, whether the thermosensitive properties of the antennal sensory neurons have any influence over mosquito thermotaxis behavior has not been explored. An early study showed that removing the antenna in *Aedes aegypti* had minimal impact on the ability to seek human odor and heat, unless more than 10 distal segments were removed (*53*). Similarly, removal of six distal segments of the antenna in *Aedes albopictus* had little effect on blood-feeding on a human arm (*54*). In our study, we found no evidence that the *Aedes aegypti* antenna distal segment is required for heat-seeking using our behavioral assay or elicit strong neural activation to heat as measured by genetically encoded calcium sensors.

Our finding contradicts previous electrophysiological studies reporting heat-evoked responses in antennal tip neurons. We speculate that this discrepancy could arise from differences in heat stimulus delivery methods. While our study used humidified, carbon-filtered air and maintained a constant flow rate throughout the trial, prior studies used dry air and did not match flow rates between the two ports delivering ambient and heated air (*50*, *76*). Another study used infrared radiation to control sample temperature in ambient air (*51*). Differences in neural activity measurement may also contribute to the discrepancy, as we used functional imaging with genetically encoded calcium indicators, while previous studies used electrophysiological methods. Functional imaging offers the advantage of observing activity across large populations of neurons in response to sensory stimuli and has been widely used to uncover the thermosensory properties, particularly in *Drosophila melanogaster* and *C. elegans*. A recent study used this technique to demonstrate humidity- and cooling-evoked neuronal activity in the antenna of *Aedes aegypti* (*25*). Building on this prior work, our study used this approach in *Aedes aegypti* to investigate heat-evoked activity in mosquito sensory appendages, in which we observed negligible heat-evoked neural activation of antenna tip sensory neurons. In line with recent work describing the role of cooling detection in thermotaxis behavior in both *Anopheles gambiae* and *Aedes aegypti* (*24*, *25*), we speculate that the antenna is tuned to detect relative decreases in convective air temperature, while leg neurons are responsible for detecting convective and conductive heat.

Recent work demonstrated that infrared light, a key component of thermal stimuli, activates mosquito antenna nerves through the genes *op1*, *op2*, and *TrpA1* (*77*). Moreover, mutant mosquitoes lacking two of these opsin genes or *TrpA1*, as well as mosquitoes with the antenna tip removed, showed a marked reduction in infrared light–seeking behavior, underscoring the critical role of infrared light in effectively guiding mosquitoes to humans. In our current work, we did not specifically focus on infrared light as an isolated thermal stimulus but rather examined a scenario where all modes of thermal heat transfer—infrared light, convective, and conductive heat—were present to the mosquitoes. In our behavioral assay, mosquitoes with the antenna tip removed continued to show strong attraction to warm surfaces, indicating that mosquitoes can use any available thermal information to maintain their strong attraction toward human-relevant skin temperatures.

### *Aedes aegypti* tarsi function as heat sensors

Insect legs are among the primary sites for taste detection. In *Drosophila melanogaster*, tarsal sensory neurons detect various nonvolatile chemosensory cues, including sugars, bitters, salts, fatty acids, water, pH, and pheromones (*39*, *40*, *58*, *78*). In *Aedes aegypti*, tarsal neurons detect water and salt to assess oviposition sites and respond to DEET contact repellency (*42*, *79*, *80*). In this study, we describe a previously unknown heat-induced activation of tarsal sensory neurons and established a role for the distal tarsal segments of the forelegs in heat-seeking behavior. Behaviorally, our findings show that removing a pair of distal tarsal segments from the forelegs, as opposed to those in the mid- or hind legs, impairs the mosquito’s ability to detect and land on heated surfaces. At the neuronal level, we demonstrate that heat activates a substantial subset of tarsal sensory neurons across the three most distal segments of both forelegs and midlegs. In addition, we provide evidence of selectively enhanced heat-evoked neuronal activity in the forelegs of *Orco* mutant mosquitoes. These findings highlight the critical role of foreleg tarsal neurons in *Aedes aegypti* thermotaxis behavior.

Removing pairs of tarsal segments from the forelegs reduced heat seeking and engorgement on the artificial blood feeder, though these behaviors were not entirely abolished. We speculate that this residual behavior may be attributed to the redundant thermosensory abilities of the neurons in the midlegs, given that these neurons also exhibited heat-evoked neuronal activity. We were unable to obtain functional imaging data from the hind leg tarsi because of high background fluorescence, leaving the role of hind legs in heat detection undetermined. In addition, this behavior may be influenced by the combined effect of increased neural activity in response to infrared light detection and convective heat sensing through decreased activity of cooling-responsive neurons at the antennal tip (*25*, *77*). Future studies that explore the combined effects of relative cooling and infrared light detection by the antenna, along with the newly identified role of the forelegs in thermosensation, will further elucidate the complex heat-detection strategies mosquitoes use to effectively identify and feed on humans.

As previous studies have shown, heat dissipates rapidly from its source (*25*, *81*). Thus, mosquitoes must approach the surface closely to detect heat and quickly assess the temperature. Given the close link between air temperature and relative humidity, we cannot fully rule out the possible influence of the moisture microenvironment on neural activity and landing behavior. The behavioral dynamics of mosquito landing events have received limited attention, and future studies using high-resolution behavioral tracking, combined with thermal and humidity monitoring, could reveal how each sensory appendage contributes to host landing behavior.

### IRs regulate mosquito thermosensitivity toward host skin temperature

Our data reveal that functional IR complexes, formed by a unique combination of IR ligand-specific and co-receptor subunits, contribute to various aspects of heat-seeking behavior. We found that *Ir25a* is required for thermotaxis behavior across all temperature ranges, suggesting that a functional IR complex containing *Ir25a* is crucial for thermosensation. In contrast, *Ir76b* mutants exhibited heightened sensitivity to milder heat while retaining wild-type responses to higher temperatures. The heightened sensitivity to temperatures below human skin temperature implies that *Ir76b*-containing IR ion channel complexes adjust the preferred temperature to a host-relevant range. The mechanism behind this adjustment remains unknown, but we speculate that *Ir76b* contributes to a thermosensitive IR complex that is insensitive to lower temperatures, and its absence lifts inhibition on the receptor complex, activating positive thermotaxis.

*Ir8a* mutants display yet another unique heat-seeking behavior, with enhanced heat seeking across the entire temperature range. This enhancement in *Ir8a* mutants resembles the behavior seen in *Orco* mutants, though it extends across a broader temperature range and is not specific to human skin temperature. *Ir8a*, like *Orco*, is selectively expressed in sensory neurons in head appendages. It will be interesting to determine whether *Ir8a* mutants also show selective up-regulation of IR subunits in the legs or they use an alternative mechanism of sensory compensation. We speculate that *Ir8a* mutants enhance heat-seeking behavior to compensate for the loss of lactic acid detection, a key component of human sweat odor. Notably, *Ir8a* mutants also show increased attraction to standing water (*82*). These findings suggest that behavioral enhancement extends beyond human-seeking behavior to include other vital activities necessary for offspring propagation. Future studies are needed to explore whether IR signaling pathways modulate additional sensory modalities.

### How is thermosensitivity enhanced in *Orco* mutant mosquito legs?

In this study, we identified *Ir140* as a critical mediator of heat-seeking behavior, as evidenced by the reduction in heat seeking in *Ir140* mutants. While *Ir140* mutants show decreased heat seeking, the behavior is not entirely abolished. This residual heat seeking could result from contributions of infrared light detection and cooling-sensing neurons at the antenna tip, but we also speculate that additional IR subunits may form functional ion channels in the forelegs that further contribute to heat detection. One of the up-regulated ligand-specific IR subunits in *Orco* mutant legs could be among the remaining genes involved in mosquito heat-seeking behavior. We speculate that *Ir140*, together with *Ir25a* and potentially other IR subunits, forms an ion channel complex within tarsal sensory neurons. This ion channel complex could serve as a direct thermosensor or function downstream in the signal transduction pathway of thermosensory neurons. Future investigation of their biophysical properties in a heterologous expression system is necessary to determine whether these ion channel complexes are intrinsically thermosensitive.

Another possible mechanism of enhanced thermosensitivity in *Orco* mutant legs could involve *Ir140*, along with *Ir25a*, modifying the ciliary membrane morphology to increase the thermosensitivity of tarsal neurons. Ciliary membrane morphology has been shown to affect neuronal function and behavior, as seen in *Drosophila* arista cooling cells, where *Ir21a* mutants failed to form the correct morphology (*35*). Future studies involving labeling of *Ir140*-expressing neurons to examine neuronal architecture, along with transmission electron microscopy to observe ciliary membrane morphology, may provide insights into the mechanism of sensory adaptation in *Orco* mutant mosquitoes. In addition, future studies will determine which modes of thermal transfer, infrared light, convective, or conductive heat are enhanced in *Orco* mutant mosquitoes.

### What are the signaling mechanisms underlying long-range sensory compensation?

Sensory compensation has traditionally associated with localized changes in synaptic connections and strength (*8*), reorganization of sensory maps (*6*), direct neuromodulation of downstream neural circuit in an opposing sensory modality (*9*), and the release of neuromodulators from remote brain structures (*10*, *11*). We believe that these mechanisms are unlikely to apply to *Orco* mutants in our study, where we report an unconventional mode of sensory compensation in which the loss of one sensory modality profoundly affects the neuronal activity of primary sensory neurons in another sensory modality.

We speculate that this form of sensory compensation arises from a non–cell-autonomous mechanism, independent of *Orco* expression in the legs. First, *Orco* expression is relatively low compared to other classes of chemosensory ion channels. Using normalized read counts from DESeq2 analysis of foreleg and midleg bulk RNA-seq, we observed very low levels of the head appendage co-receptors *Orco* (83 ± 40) and *Ir8a* (88 ± 7), compared to the IRs we speculate to play a role in heat seeking in the leg: *Ir25a* (17,841 ± 500), *Ir76b* (15,621 ± 1023), and *Ir140* (571 ± 14). Furthermore, our expression data are consistent with previous studies where *Orco*-expressing neurons were not detected in the tarsi of *A. coluzzii* (*83*), in the *Drosophila melanogaster* leg single-cell RNA-seq dataset (*84*), or in the ventral nerve cord innervations by *Orco*-expressing peripheral sensory neurons in *Drosophila melanogaster* (*27*). These findings support the hypothesis that *Orco* contributes to thermosensory compensation through non–cell-autonomous mechanisms.

### Future directions aimed at addressing the limitations of our study

Although we provide evidence of long-range sensory compensation, our experiments do not directly address the mechanism by which it forms. One potential mechanism involves long-range excitability changes due to neuromodulation. Our RNA-seq data reveal enrichment of up-regulated neuromodulatory receptor gene expression in the legs of *Orco* mutants, notably receptors involved in hunger and metabolism (fig. S6). This suggest a possible mechanism where neuromodulation of leg sensory neurons, potentially through circulating hemolymph, induces transcriptional changes and up-regulation of thermosensors to enhance heat sensitivity in response to the absence of olfactory inputs. Another potential mechanism is through direct neural modulation by *Orco*-expressing neurons of tarsal taste neurons. *Orco* is primarily expressed in head sensory appendages, with projections primarily to the antennal lobe and also to the subesophageal zone (*26*, *27*, *85*, *86*). Tarsal sensory neurons project not only to the ventral nerve cord but also to the subesophageal zone (*80*, *87*–*89*). Thus, the subesophageal zone may serve as an integration center, acting as the potential site of direct modulation of *Ir140*-expressing thermosensory tarsal neurons by *Orco*-expressing neurons.

Because our functional imaging data involve examining detached legs in *Orco* mutants, acute neuronal modulation is unlikely to be happening. One future approach to investigate the possible role of neural activity in sensory compensation would be to acutely silence all *Orco*-expressing neurons in head appendages and ask whether sensory compensation emerges in foreleg sensory neurons. Another possibility is that these changes occur during development because the *Orco* mutant is a constitutive knockout, leading to a loss of *Orco* across all mosquito life stages. Early loss of *Orco* expression and function could have a lasting impact on the identity and properties of leg sensory neurons. It is noteworthy that *Orco* expression has been detected during the early embryonic stage (*90*), and transcriptomic analyses suggest a regulatory role for *Orco* in ion channel signaling in *Aedes aegypti* embryos (*91*). With the increased sophistication of genetic tools in the mosquito, it may be possible in the future to knock out the *Orco* gene acutely in adult mosquitoes to determine whether sensory compensation in the leg occurs and over what time frame.

## MATERIALS AND METHODS

### Human and animal ethics statement

Blood-feeding procedures and mosquito behavior with live human hosts were approved and monitored by The Rockefeller University Institutional Review Board (IRB protocol LVO-0652) and the Rockefeller University Institutional Animal Care and Use Committee (IACUC protocol 20068-H). Human participants gave their written informed consent to participate in this study.

### Mosquito maintenance and generation of experimental crosses

Mosquito strains were reared at 26°C with 70 to 80% relative humidity and daily 14-hour light and 10-hour dark cycle (lights on at 7 a.m.) as previously described (*20*). All behavioral experiments except the thermal animal tracking assay were performed at this temperature. Embryos were hatched in a hatching broth media, which was made by crushing one pellet of fish food (TetraMin Tropical Tablets) using a mortar added to 850 ml of deionized water and further autoclaved. The following day, 2500 ml of deionized water was added to the hatched larvae, and they were fed two fish food tablets per day until pupation. After eclosion, adult mosquitoes had access to 10% sucrose (w/v in distilled water), which was delivered in a Boston clear round 60-ml glass bottle (Fisher Scientific, FB02911944) with the sugar dispensed through a saturated cotton dental wick (Richmond Dental, 201205). Female mosquitoes were blood fed on mice or human arms for egg collection. Eggs were dried at 26°C and 70 to 80% humidity for 3 days and then stored at ambient temperature and humidity for up to 4 months. All behavior experiments were carried out using adult female mosquitoes that were 7 to 21 days after eclosion in the light phase of the photoperiod. The *Aedes aegypti* Orlando strain was used as the wild-type control and in the generation of all animal crosses except Fig. 1 (E and G), where the genetic background was the Liverpool IB12 strain. We used heteroallelic null mutant mosquitoes in our experiments to eliminate any possible off-target genetic effects or behavioral artifacts due to laboratory selection (except for the *Orco* allele in the double-mutant experiment shown in Fig. 9). These experimental strains were generated by crossing two independently isolated and maintained mutant lines with their respective heterozygous genetic controls for all our experiments.

### Manipulation of sensory appendages

Adult female mosquitoes were immobilized using cold anesthesia and subsequently positioned on a chilled cold block. Sensory appendages were trimmed with 2.5-mm straight-edge Vannas spring scissors. For experiments involving the trimming of antennal tips, the three most distal antenna segments (segments 11 to 13) were removed from both antennae. When conducting full antenna removal experiments, both antennae were severed right above the Johnston’s organ. For tarsal tip cut experiments, the third, fourth, and fifth tarsomeres were removed from each pair of forelegs, midlegs, and hind legs. Mosquitoes were allowed to recover overnight at 26°C with 70 to 80% relative humidity. Mock-treated controls underwent cold anesthesia, and the sensory appendages were handled with the Vannas spring scissors, but no trimming was performed.

### Arm-next-to-cage assay

This assay was modified from an arm-next-to-cage assay described previously (*92*). Briefly, for each trial, 20 to 40 adult female mosquitoes were sorted under cold anesthesia (4°C) and transferred to a 28-cm^3^ cage and allowed to recover overnight at 26°C with 70 to 80% relative humidity and fasted by replacing 10% sucrose with deionized water. A human forearm was placed adjacent to one side of the cage, separated by two 10-ml serological pipet tips, and perpendicularly to the arm. This arm positioning allowed mosquitoes to detect human odor, CO_2_, and heat while preventing the mosquitoes from directly making skin contact. A monochrome complementary metal-oxide semiconductor (CMOS) camera (The Imaging Source, DMK37BUX178) with a 12-mm/F1.8 lens (Edmond Optics, no. 33-303) was positioned to take images of mosquitoes responding to the human arm. Trials ran for 5 min, and images were acquired once every 5 s using the IC Capture Image Acquisition software (The Imaging Source). We manually counted the number of mosquitoes resting overlaying the human arm to quantify mosquito responses. For the human arm experiment with DEET, 10% DEET (*N*,*N*-diethyl-3-methylbenzamide; Sigma-Aldrich D100951, mixed with 100% ethanol, v/v) was applied on the entire length of the forearm surface facing the cage. The applied 10% DEET was allowed to dry for 10 min before each trial. After drying, behavior experiments were performed as described above.

### Nylon-next-to-cage assay

This assay is a modified version of the arm-next-to-cage assay as above. Instead of a live human arm, a clear 80-well microcentrifuge test tube rack was inserted into a worn or unworn nylon sleeve. Human odor collection using nylon sleeves was described previously (*36*). The nylon sleeves were kept taut using binder clips. This nylon sleeve stimulus was further mounted to a ring stand and placed adjacent to one side of the cage, ensuring it did not make direct contact with the cage. An 80-well microcentrifuge test tube rack was used to mimic the surface area of the human arm that mosquitoes encountered during the arm-next-to-cage assay (6.5 cm by 22.5 cm surface). Image acquisition was performed as described in the arm-next-to-cage assay.

### CO_2_ activation assay

Twenty to 40 adult female mosquitoes were sorted under cold anesthesia (4°C) and transferred to a 28-cm^3^ cage and allowed to recover overnight at 26°C with 70 to 80% relative humidity and fasted by replacing 10% sucrose with deionized water. Sorted mosquitoes were transferred into a custom-made plexiglass box (30 cm^3^), with carbon-filtered air pumped continuously into the box via a diffusion pad installed on the ceiling of the enclosure. Mosquitoes were allowed to acclimate for 10 min. Following acclimation, a 20-s pulse of CO_2_ was applied, and flying activity was monitored for 10 min. A monochrome CMOS camera (The Imaging Source, DMK37BUX178) with a 12-mm/F1.8 lens (Edmond Optics, no. 33-303) was positioned to acquire images at one frame per second using the IC Capture Image Acquisition software (The Imaging Source) of the entire behavior box to capture of mosquitoes flying in response to CO_2_.

### Heat-seeking assay

Experiments were performed as previously described (*13*, *29*). Briefly, 40 to 50 adult female mosquitoes were sorted under cold anesthesia and allowed to recover overnight at 26°C with 70 to 80% relative humidity and fasted by replacing 10% sucrose with deionized water. Before each trial, sorted and fasted mosquitoes were transferred into a custom-made plexiglass box (30 cm^3^), with carbon-filtered air pumped continuously into the box via a diffusion pad installed on the enclosure’s ceiling, and each stimulus period lasted 3 min on a single Peltier element (6 cm by 9 cm, Tellurex). The surface was covered with 15 cm by 17 cm standard white letter-size printer paper (NMP1120, Navigator) and held taut by a magnetic frame. CO_2_ pulses (20 s, to >1000 parts per million above background levels) were added to the air stream and accompanied all heat stimulus period onsets. Each heat stimulus lasted 180 s, with a 9-min interval between heat stimuli. Mosquito landings on the Peltier were monitored by fixed cameras (FFMV-03M2M-CS, Point Grey Research), with images acquired at one frame per second. A custom MATLAB script was used to control the CO_2_ pulse and Peltier set-point temperature, as well as for image acquisition. The Peltier temperature was measured using a thermocouple probe embedded in the Peltier element, and we report the Peltier set-point temperature instead of surface temperature throughout the paper as we believe it is the most reliable and consistent metric across experimental trials. Images were analyzed using custom MATLAB scripts to count mosquito landings within a fixed target region corresponding to the Peltier area. Mosquito occupancy on the Peltier was quantified by calculating the percentage of mosquitoes within the target region for each frame and then averaging this percentage over seconds 90 to 180 of each stimulus period. The data are expressed as “% on Peltier.”

### Heat-seeking assay with DEET

To test whether DEET could disrupt mosquito attraction without host odor, we tested the female mosquito’s thermotaxis to a 40°C Peltier element in the presence or absence of DEET. A standard 28-cm^3^ rearing cage (Bioquip) was placed inside a clear vinyl bag. The bag and cage were positioned adjacent to a polymerase chain reaction (PCR) machine, so the Peltier element was ~1 cm from the cage screen. A Firefly MV camera was positioned to take stereotyped images of mosquitoes responding to the heat source (Point Grey Research). A Flypad (Flystuff.com) was placed inside the bag, on top of the cage, as a source of carbon-filtered air and CO_2_. The assay was assembled on a metal peg board to maintain the fixed position of the components: a rearing cage inside a vinyl bag, a camera, and a PCR machine. Ten to 30 min before the start of the assay, 25 adult female mosquitoes were released into each cage to acclimate. Before starting each trial, 100 μl of 100% ethanol or 10% DEET in ethanol was applied to a 2 cm by 6 cm strip of Whatman filter paper (GE Healthcare). The treated paper was hung from a rectangular plastic frame in front of the Peltier element so it was not in contact with the cage screen. For each trial, carbon-filtered air was pumped into the assay for 60 min to remove the host odor. After the 60-min prestimulus period, 10% CO_2_ was released using the Flypad while the Peltier element was heated to 40°C. For each 5-min trial, images were taken every 10 s. After each trial, the mosquitoes were placed under cold anesthesia and counted to verify the number used. To quantify mosquito behavioral responses in this assay, we counted the number of mosquitoes on the cage screen near the Peltier element. Each data point represents a percentage of mosquitoes attracted to the heat source, which was determined by calculating the average number of mosquitoes from the last 10 images of each trial divided by the total number of mosquitoes in the assay, multiplied by 100. All scored images were cropped to the same size with Fiji software using a macro [makeRectangle(269, 176, 347, 237); run(“Crop”); run(“Save”); run(“Open Next”)]. Once cropped, images were visually scored with assistance from the multipoint counting tool in Fiji.

### SciTracks assay

The assay was performed as previously described (*13*). A multi-insect three-dimensional (3D) tracking system was custom designed and built in collaboration with SciTrackS GmbH (Sci-Trak). It consists of a flight chamber (1.25 m wide by 1 m high by 0.75 cm deep) with clear acrylic sides and front. A pump continuously provides carbon-filtered air (Cole-Parmer Quiet Pressure Pump, no. 79610-81). CO_2_ can be added to this airstream through the use of a computer-controlled solenoid valve, a valve controller (NResearch Inc., model 360D1X75R) operated by a MATLAB script through an analog/digital interface (Measurement Computing Corporation, model USB-1208FS). Humidity was controlled using a closed-loop humidification system consisting of an ultrasonic humidifier (SPT, model SU-1051B) controlled by a temperature and humidity probe attached to a microcontroller. Humidity remained at 45 ± 4% for the duration of each trial. Tracking is accomplished by imaging the chamber with two offset cameras (Basler piA640-210gm; Basler AG, Ahrenburg, Germany with 1/2 4- to 12-mm F/1.2 IR Aspherical objects, Tamron, Commack, NY, USA). Lighting is provided by two banks of IR light-emitting diodes (LEDs) (Metaphase model ISO-23-IRN-24-AL2, Metaphase Technologies, Bensalem, PA). 3D position information of each visible mosquito was calculated in real time at 200 Hz using custom-built software based on previously published algorithms (*93*). Tracks were further processed, filtered, and analyzed using MATLAB. For each trial, 20 female mosquitoes (5 to 10 days old, fasted overnight with access to water) were added to the flight chamber with a mechanical aspirator. After acclimation and tracking baseline activity for 30 min, a 40-s pulse of 100% CO_2_ was added to the airstream at a flow rate of 1360 ml/min, which raised the concentration of CO_2_ to ~1%, as measured in the middle of the flight chamber. (CARBOCAP Hand-Held Carbon Dioxide Meter GM70, Vaisala Inc., Woburn, MA, USA). Tracking continued for another 20 min to measure activity in response to this pulse of CO_2_. Data are presented as the population distance tracked in 10-s bins or as cumulative distance tracked per mosquito in the 6 min immediately before or after CO_2_ addition.

### Optothermocycler assay

The optothermocycler assay was performed as previously described (*56*). The assay was constructed on top of a PCR thermocycler (Eppendorf Mastercycler) using optomechanical components (Thorlabs). Green light at a wavelength of 530 nm was emitted using six Luxeon Star SP-01-G4 LEDs under the control of an Arduino Uno board. The surface of the PCR block was covered with black tape to minimize glare (Thorlabs, T137-2.0). Temperature monitoring was conducted through a type T thermocouple (Harold G Schaevitz Industries LLC, CPTC-120-X-N), which was connected to an Arduino board (Arduino, A000066) via a thermocouple amplifier (Adafruit, MAX31856). The thermocouple sensor was securely positioned on the lower right surface of the PCR block using black tape (Thorlabs, T137-2.0). Using a custom Processing script, temperature measurements and light output were logged at 100-ms intervals through the Arduino. The video was synchronized with the light and temperature stimuli by using an infrared 940-nm LED (Adafruit, 387) positioned within the camera’s field of view. Mosquitoes were illuminated with an 850-nm infrared LED strip (Waveform Lighting) surrounding the mosquitoes’ plate orthogonal to the camera’s view. The video was recorded using a Blackfly camera (FLIR BFS-U3-16S2M-CS) outfitted with a 780 nm long pass filter (Vision Light Tech, LP780-25.5) at 30 frames per second using Spinview software (Teledyne FLIR). The PCR thermocycler was programmed to apply heat stimuli directly from ambient to a noxious temperature range of 24° to 60°C.

### Arm feeding assay

Female adult mosquitoes were cold anesthetized, and 10 to 20 mosquitoes were sorted into a behavioral container made from a 32-ounce (946 ml) HDPE plastic cup (VWR, no. 89009-668). The behavioral container was prepared by cutting a 10-cm hole in the lid with a razor blade and covering the cup with a 20 cm by 20 cm piece of white 0.8-mm polyester mosquito netting (American Home & Habit Inc., no. F03A-PONO-MOSQ-M008-ZS) and securing the mesh to the cup using the modified lid. Sorted mosquitoes were allowed to recover overnight at 26°C with 70 to 80% relative humidity and fasted by replacing 10% sucrose with a cotton wick saturated with deionized water placed on top of the container across the mesh netting. For each experimental trial, mosquitoes were allowed to acclimate for 10 min, and a live human arm was placed on top of the container. Mosquitoes were allowed to feed on the human arm through the mesh netting for 10 min. They were then anesthetized at 4°C and scored as fed if any level of feeding was observed, as assessed by visual inspection of the abdomen of the animal.

### Glytube feeding assay

Female adult mosquitoes were sorted into individual behavioral containers as described with the arm feeding assay. The assay chamber was a modification of previously published methods (*61*) and used a translucent polypropylene storage box 36 cm L by 31 cm W by 32 cm H with a removable lid. One 1.5-cm hole was made on the chamber wall and was used to introduce silicone tubing for CO_2_ delivery. The CO_2_ diffusion pad (8.9 cm by 12.7 cm; Tritech Research) was affixed to the inner center of the lid to allow delivery of purified air and CO_2_ to condition the chamber atmosphere during the trial. Females were fed sheep blood (Hemostat, DSB100) supplemented with adenosine triphosphate (ATP) (Sigma-Aldrich, A6419) at a final concentration of 1 mM using a Glytube membrane feeder (*61*). All blood meals and Glytubes were preheated for at least 10 min in a 45°C water bath, and ATP was added to meals immediately before feeding. At the start of each trial, up to four cups were placed in the assay chamber, and the animals were allowed to acclimate for 10 min before one Glytube containing 1.5 ml of sheep blood + ATP mixture was placed on top of each cup, and CO_2_ was turned on for 10 min. Mosquitoes were then anesthetized at 4°C and scored as fed if any level of feeding was observed, as assessed by visual inspection of the abdomen of the animal.

### RNA extraction and sequencing

Seven- to 10-day-old female wild-type and *Orco^5/16^* mosquitoes were cold anesthetized and kept on ice for up to 30 min or until dissections were complete. Forelegs and midlegs were dissected and collected on ice in a 1.5-ml tube. Five samples from each genotype were collected, and each replicate consisted of legs from 20 animals. After collecting each sample, tubes were immediately snap frozen using a cold block (Simport, S700-14) chilled with ethanol containing dry ice. Dissected tissue was stored at −80°C until tissue collection was complete. RNA was extracted by homogenizing each sample in TRIzol reagent (Invitrogen, 15596026) using a handheld homogenizer (DWK Life Sciences, 749540-0000). To the homogenized sample, 20% of the total TRIzol regent volume of chloroform:isoamyl alcohol (Fisher Scientific, AC327155000) was added for phase separation. The aqueous phase containing total RNA was transferred into a fresh ribonuclease-free tube and cleaned using the PicoPure Kit (Thermo Fisher Scientific, KIT0204) following the manufacturer’s instructions, including the deoxyribonuclease treatment step. Samples were run on a Bioanalyzer RNA Pico Chip (Agilent, 5067–1513) to determine RNA quantity and quality. Libraries were prepared using the Illumina TruSeq Stranded mRNA kit (Illumina, 20020594). Samples were pooled, and sequencing was performed at The Rockefeller University Genomics Resource Center on an Illumina NovaSeq 6000 sequencer using V1.5 reagents, the SP flow cell, and NovaSeq Control Software V1.7.0 to generate 150-bp paired-end reads. All samples were sequenced on the same flow cell to correct for potential batch effects. Data were demultiplexed and delivered as fastq files for each library. Sequencing reads have been deposited at the National Center for Biotechnology Information (NCBI) Sequence Read Archive (SRA) under BioProject PRJNA1020561.

### Transcriptome alignment, quantification, and differential gene expression analysis

The sequencing data were aligned and quantified with the Salmon quantification software (*94*) using the reference transcriptome generated from *Aedes aegypti* LVP_AGWG (https://vectorbase.org/vectorbase/app/record/dataset/DS_cc8d875d2e) genome and the updated version of the previously published gene annotation file from Jové *et al.* (*61*). The most current version of the reference transcriptome is available at https://github.com/VosshallLab/Morita_Vosshall2023. Gene expression levels as raw read counts and abundances were retrieved using tximport (version 1.22.0) (*95*). The raw read counts and differential expression analysis were normalized using DESeq2 (version 1.34.0) (*96*). The FDR was calculated using Benjamini and Hochberg multiple testing correction, using the FDR cutoff at 0.01. Foreleg, midleg, and hind leg samples from the neurotranscriptome dataset were reanalyzed following the above steps (*30*). Leg samples from sugar-fed and ovipositing samples were used for analysis (SRR1167521-533, SRR1166473-484, SRR1167469-477, SRR1167554-562, and SRR1167491-492). A pairwise differential expression analysis using DESeq2 was performed. The FDR was calculated using Benjamini and Hochberg multiple testing correction, using the FDR cutoff at 0.01.

### Genome alignment and visualization

The sequencing reads were first processed using Trim Galore (version 0.6.7) to apply library adapter and quality trimming (https://github.com/FelixKrueger/TrimGalore). Trimmed reads were aligned to the *Aedes aegypti* LVP_AGWG genome using STAR aligner (version 2.7.6a) (*96*). Aligned reads were visualized using IGV (*98*).

### Visualization of reporter line–expressing cells in tarsus and cell counting

We visualized sensory neurons in the fifth tarsomere of the tarsus as previously described (*80*). We used the following reporter lines *brp-T2A-QF2w* (*60*), *Ir25a-T2A-QF2* (*26*), and *Ir76b-T2A-QF2* (*26*) crossed to *15xQUAS-dTomato-T2A-GCaMP6*s (line no. 5) animals (*80*). *Gr4-T2A-QF2* (*61*) was crossed to *15xQUAS-dTomato-T2A-GCaMP6*s animals (line #1) (*61*). Animals were anesthetized on wet ice. Entire tarsi were removed and placed in ice-cold ethanol for ~20 s and then mounted onto a prepared slide with 50 μl of glycerol pipetted using a 200-μl pipette tip with the end cut to prevent bubbles. A 1.7-mm coverslip was mounted, and pressure was gently applied before sealing with nail polish. Confocal z-stacks were acquired on a Zeiss Inverted LSM 880 laser scanning confocal with a 40×/0.8 numerical aperture water immersion corrected objective at a 1024 × 1024 pixel resolution and a voxel size of 0.2076 μm by 0.2076 μm by 1 μm. Cranial and caudal neuron layers were acquired in the order of proximity to the objective, which was random on the basis of the leg’s orientation. Cranial and caudal layers were identified on the basis of whether the leg imaged was the left or right leg and relative to the position of the claw. The cranial and caudal layers are easily distinguished by a space between the cranial and caudal layers that rarely, if ever, contains labeled neurons and also includes the trachea. Whichever layer was further away from the objective was typically dimmer. Laser intensity correction was applied to ensure the distal layer received enough laser power, and the proximally imaged layer was not saturated. Cells were counted using Imaris software (Bitplane). The 3D image was cropped for each image to include the entire fifth tarsomere. Cells were counted first using an automated counting algorithm, and then each cell was confirmed manually. The estimated spot diameter for the automated counter was 5 μm. All cells were counted for each collected sample. For distinguishing cell counts in cranial and caudal layers on an individual sample or counting for 50-μm distal-proximal segments of the fifth tarsomere, clipping planes were used on the 3D image so that no individual cells in a given sample were counted twice. Fluorescent sensilla counting was done manually. The side view of the cranial and caudal layers of the tarsus was achieved by rotating a 3D image of a confocal z-stack.

### Ex vivo sensory appendage imaging

Calcium imaging was performed on a microscope setup previously described (*61*). Experiments were performed on an inverted Ti-2E wide-field microscope (Nikon) with a dual fluorescein isothiocyanate/tetramethyl rhodamine isothiocyanate band-pass scube and alternating emission wheel with 520/40 green fluorescent protein and 628/40 red fluorescent protein (RFP) band-pass filters. An nd2 filter was added with the 628/40 RFP band-pass filter to attenuate the dTomato signal. Images were acquired with a 20×/0.75 air objective (Nikon) and Zyla 4.2 Plus camera. Calcium imaging experiments were performed on female mosquitoes 7 to 14 days after eclosion. The imaging chamber was prepared by affixing a Gold Seal Cover Glass, No. 1 22 mm by 40 mm coverslip (Ted Pella, no 260353) to a low-profile open diamond bath imaging chamber (Warner Instruments, RC-26GLP) using a silicone lubricant (Dow Molykote 111 O-Ring Silicone Lubricant). Female mosquitoes were anesthetized briefly at 4°C for dissection. Each dissected sensory appendage was removed using fine forceps and Vannas spring scissors. Dissected sensory appendages were placed on a 15-mm round cover glass (Warner Instruments, CS-15R) using a very thin layer of silicone lubricant. This sensory appendage preparation, along with the coverslip, was directly secured inside the imaging chamber. dTomato fluorescence was examined before each trial to locate and focus on the sensory appendage intended for imaging. During the experiment, each image acquisition step captured one GCaMP image and one dTomato image, separated by less than the 100 ms required to switch the emission filter wheel. Image acquisition was triggered at approximately one frame per second for each channel.

### Stimulus delivery and monitoring

Temperature stimulus was delivered to the dissected sensory appendage using a continuous flow of carbon-filtered air supplied by an air pump (Welch, 5078S). The air was passed through two rounds of deionized water using a gas washing bottle (Chemglass, CG111206) to filter and moisten the air stimulus. This main air stimulus was further bifurcated into two independent air lines, and the opening/closing of each opening was controlled using its dedicated solenoid valves (Warner Instruments, VCS-8-PTFE), one for ambient air and another for heated air. Each air line was passed through an independent flow meter to match the flow rate between the two air lines. An ambient temperature air line outlet was placed adjacent to the dissected sensory appendages. This ambient air line functioned as the default air line and was kept open at the beginning and the end of each experiment. As for the heated air line, tubing was connected to a dual in-line solution Peltier heater/cooler device (Warner Instruments, SC-20), and the outlet was placed directly above the sensory appendage to be imaged. The air temperature was adjusted by controlling the Peltier heater/cooler device temperature using an external bipolar temperature controller (Warner Instruments, CL-100). By default, this heated air line is kept closed unless a heated stimulus is applied to the sensory appendages. The experiment started with the ambient air line opened and the heat air line closed. The ambient air line was closed for each heat stimulus while the heat air line was opened. When the sample temperature reached ~33°C, the ambient air line was opened while the heat air line was closed. The opening of each air line was controlled through analog signals provided by the image acquisition software. For the slow heat ramp protocol used in Fig. 7), the temperature stimulus was delivered to the dissected sensory appendage through a single air line with an air source as previously described. This air line was passed directly through the Peltier device, where the temperature can be changed throughout the experiment. At the beginning of each trial, the Peltier device was set at 20°C for ambient air temperature delivery. During the experimental trial, the Peltier temperature was changed to 50°C, so the target temperature that the sensory appendages were experiencing was around 33°C. Once the temperature reached our target temperature, the Peltier temperature was switched back to 20°C. To monitor the temperature of the sensory appendages, a thermocouple microprobe was placed adjacent to the samples (Physitemp, IT-23) and was recorded using a digital thermometer (Physitemp, BAT-12) during the entire experiment. After each experimental trial, tissue samples were immersed in physiological saline, and the imaging field of view was refocused. To validate neuronal health and activity, 500 mM KCl was perfused over the tissue samples, and images were acquired and analyzed as described above. Tissue samples with response rate >90% were further used for analysis. Samples that did not meet this threshold for viability were exceedingly rare, indicating that these explant imaging preps preserve cellular function (antenna, 0 of 9 trials below threshold; maxillary palp, 0 of 8 trials below threshold; proboscis, 1 of 10 trials below threshold; legs, 3 of 32 trials below threshold).

### Calcium imaging data analysis

Fluorescent intensities emitted by fluorescent proteins are temperature dependent and exhibit an inverse relationship with temperature (*99*, *100*). To account for this biophysical property of fluorescent proteins, we took advantage of animals coexpressing GCaMP6s and dTomato in the same cell by capturing images at both fluorescent channels. dTomato functioned as a reference imaging channel to correct for both movement artifacts and temperature-induced changes in fluorescence intensity. Regions of interest (ROI) were marked using the dTomato signal captured at the beginning of each experiment. ROIs were drawn around individual dTomato-positive neurons. ROI information was overlaid with all image frames captured during each experimental trial, and fluorescent intensities were recorded for each time point from both GCaMP and dTomato imaging channels. The ratio of GCaMP and dTomato was used to report the temperature-dependent changes in fluorescent intensities at each time point and was normalized to the first frame to report the data as Δ*R*/*R*.

### *Ir140* crRNA design

*Ir140* single and *Orco, Ir140* double mutants were generated using methods described previously (*21*). Single-guide RNA (sgRNA) sequences were designed with the CHOPCHOP sgRNA design tool (https://chopchop.cbu.uib.no/) (*101*) using the following parameters: In, *Aedes aegypti* (AaegL5.0); Using, CRISPR-Cas9 with the *Ir140* CDS sequence (NM_001358695.1) as the target sequence. For each type of *Ir140* mutant, pairs of target sequences were selected for targeted double-stranded break-induced mutagenesis, with each pair flanking roughly 250 bp. The following sequences were used for each type of *Ir140* mutant generation:

*Ir140* single mutant:

1) CAGAAGTTATCATAGCACCTGTTTTAGAGCTATGCT

2) TCTCCATCATGCCAACACACGTTTTAGAGCTATGCT

*Orco^16^, Ir140* double mutant:

1) CCGCCTTCATAATCAATCAGGTTTTAGAGCTATGCT.

2) CTCATCGTGATCTACTCTGAGTTTTAGAGCTATGCT.

*Orco^5^, Ir140* double mutant:

1) CCGCCTTCATAATCAATCAGGTTTTAGAGCTATGCT.

2) CTCATCGTGATCTACTCTGAGTTTTAGAGCTATGCT

### CRISPR injection mix

Two microliters of 100 μM Alt-R trans-activating CRISPR RNA (tracrRNA, IDT, no. 1072532) was mixed with 1 μl of each custom-designed Alt-R CRISPR RNA (crRNA, IDT, 100 μM) and 1 μl of Nuclease-Free Duplex Buffer (IDT, no. 11-01-03-01). This initial mix was then incubated at 95°C on a thermocycler for 5 min, followed by 5 min at room temperature. After both incubations, 3 μl of Alt-R S.p. Cas9 V3, glycerol-free, at 1 μg/μl (IDT, no. 10007806) and 2 μl of nuclease-free water were added. The solution was incubated at room temperature for 5 min and then placed on ice until the time of injection.

### Injection procedure

Mosquito embryos were injected in-house using a Sutter Instrument injection system (Product no. FG-BRE, FG-MPC385) and a Zeiss SteREO Discovery stereo microscope (no. 495007-9880-010). Embryo injection was carried out as previously described (*102*). About 100 eggs were injected per injection session 60 min after laying them. A prepulled (Sutter Instrument, no. FG-P1000) and prebeveled (Sutter Instrument, no. FG-BV10-D) aluminosilicate glass with Filament (Sutter Instrument, no. AF100-64-10) was loaded with the injection mix using a Microloader tip (Eppendorf, catalog no. 930001007). The eggs were injected on their posterior side and visually examined for slight expansion during injection to endure the transfer of the injection solution. In total, approximately 505 embryos from wild-type *Aedes aegypti* Orlando strain were injected for the generation of *Ir140* single-mutant strains, while 490 *Orco^5/5^* and 510 *Orco^16/16^* embryos were injected for the generation of *Orco, Ir140* double mutants. After each injection session, the eggs were recovered from the slide and placed on wet filter paper in a cup with wet cotton on the bottom to maintain humidity during recovery. The cup was covered with tissue paper and kept for 3 days in the insectary (27°C, 80% relative humidity) until the eggs were transferred to a hatching solution.

### Screening the injected lines

The hatching rate following injection was ~2 to 20%. G_0_ females from each injection were crossed with males from their respective background strains, and their G_1_ offspring were screened for germline mutation by PCR amplification and Sanger sequencing of the regions flanking the sgRNA target sites. Two unique stable mutant lines, each resulting in an early stop codon due to a frameshift mutation, were isolated from each injection except for the *Orco^5^* injection, which had a low hatching rate, and we did not find any isolates with signs of gene modification. For the *Ir140* single mutant, one isolated mutant line had a 144-bp out-of-frame mutation (*Ir140^144^*), and the other had a 17-bp insertion (*Ir140^17^*). For *Orco^16^, Ir140* double mutant, one isolated mutant line had a 49-bp deletion (*Ir140^49^*), and the other had a 14-bp deletion (*Ir140^14^*). Virgin females from each mutant line were backcrossed to their respective genetic background males for three generations before establishing stable homozygous lines. Homozygous mutant lines were intercrossed to generate heteroallelic mutants tested in all behavior experiments alongside appropriate genetic controls.

### *Ir140* mutant genotyping

Genotypes were confirmed using Phire Tissue Direct PCR Master Mix (Thermo Fisher Scientific, F170L) followed by gel electrophoresis and Sanger sequencing (Genewiz) using the following primer combination for each mutant allele:

*Ir140^144^* and *Ir140^17^*single mutants:

Forward: ACGCCACACCCGTTCAAATC

Reverse: CGATTTGACTAGCTCCGGAATTG

*Orco^16^, Ir140^49^* double mutant:

Forward: TGCTAAGCCACATCAACAAG

Reverse: ACGTAGTTGAAGCGCCATTTG

*Orco^16^, Ir140^14^* double mutant:

Forward: AGCCGGTCGTCAATCATTAC

Reverse: ACGTAGTTGAAGCGCCATTTG.

The *Ir140^144^* single-mutant allele was detected by a 144-bp deletion using standard electrophoresis protocol (wild-type band: 515 bp; *Ir140^144^* band: 317 bp), while *Orco^16^, Ir140^49^* double-mutant allele was detected by a 49-bp deletion on a 2% agarose gel with a low-voltage (90 V) electrophoresis protocol (wild-type band: 250 bp; *Orco^16^, Ir140*^*49*^ band: 210 bp). For mutants with small deletions, the presence or absence of endogenous restriction enzyme target sites was used to distinguish between mutant and wild-type alleles. PCR products were generated and digested with the indicated enzyme, producing the indicated bands in mutant and wild-type:

*Ir140^17^* single mutant with Sty I:

Wild-type: 329 and 186 bp; *Ir140^17^*: 532 bp

*Orco^16^, Ir140^14^* double mutant with Ava II:

Wild-type: 151 and 99 bp; *Orco^16^, Ir140^14^*: 236 bp

*Orco^16^* mutant allele for *Orco^16^, Ir140^14^* and *Orco^16^, Ir140^49^* double mutants were independently validated using the following primers:

Forward: CCGCACGCTGGGCATCTGGAATC

Reverse: ACGGATAGCACTGTAGTCACCAT

All genotyping experiments were performed with a no DNA control and fragment size validation using a 1Kb Plus DNA ladder (Thermo Fisher Scientific, 10787026). See fig. S7.

### Quantification and statistical analysis

All statistical analyses were performed using R version 4.1.3 (*103*). Details of graphical representations and statistical methods are reported in the figure legends.
