# Supplementary material for: Cross-modal sensory compensation increases mosquito attraction to humans
Source: Sci Adv. 2025 Jan 1;11(1):eadn5758. doi: 10.1126/sciadv.adn5758 (PMC11691641; doi:10.1126/sciadv.adn5758)
Supplement: Supplementary file 1 — Figs. S1 to S8 Legends for movies S1 to S4 [file sciadv.adn5758_sm.pdf]

Science Advances
AAAS

Supplementary Materials for

Cross-modal sensory compensation increases mosquito attraction to humans

Takeshi Morita *et al.*

Corresponding author: Takeshi Morita, tksh.morita@gmail.com

Sci. Adv. **11**, eadn5758 (2025)
DOI: 10.1126/sciadv.adn5758

The PDF file includes:

Figs. S1 to S8
Legends for movies S1 to S4

Other Supplementary Material for this manuscript includes the following:

Movies S1 to S4

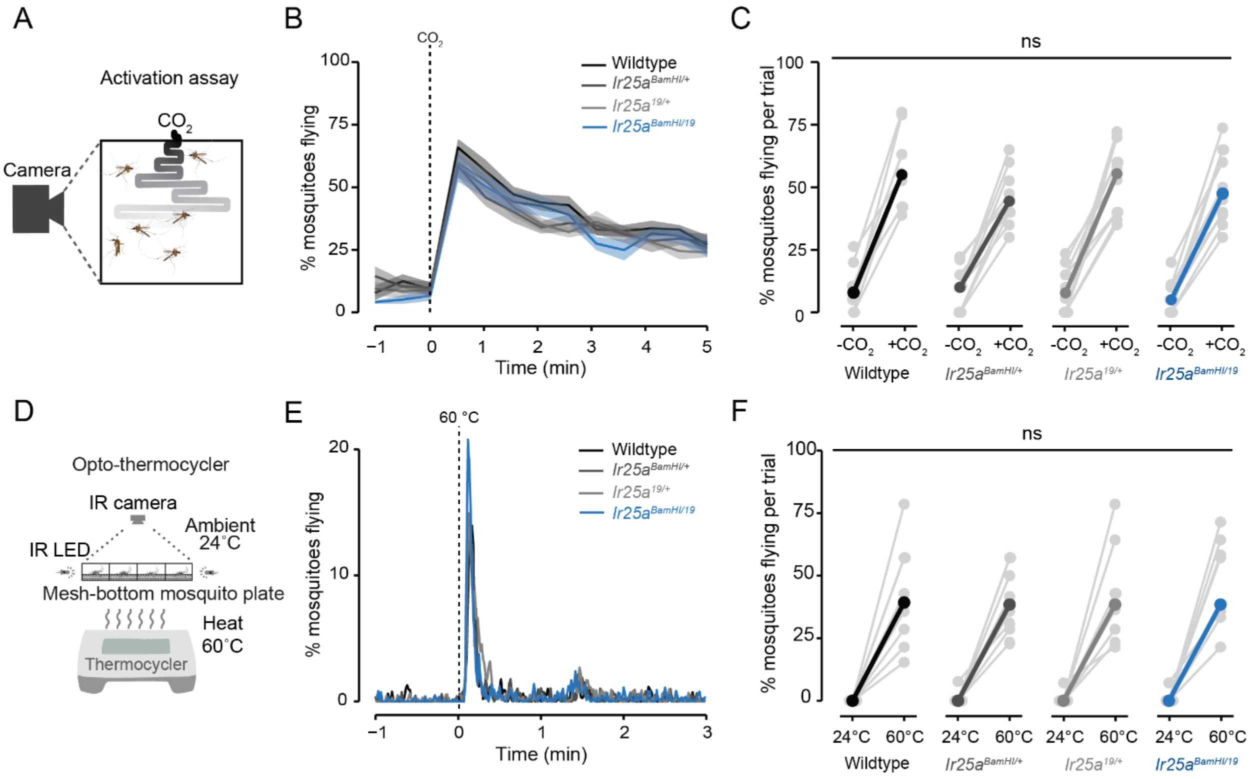

Fig. S1. – Related to Fig. 1

***Ir25a* mutants display normal CO_2 and noxious heat detection.**

(**A**) Schematic of the CO_2 activation assay. (**B**) Percent of mosquitoes flying in the CO_2 activation assay for the indicated genotypes. Data are presented as mean ± SEM (n = 8-9 trials/genotype). (**C**) Quantification of mosquitoes flying during each experimental trial before and after 1 minute after a 20-second pulse of CO_2 for the indicated genotypes (n = 8-9 trials/genotype, $p > 0.05$, between all genotypes post CO_2, one-way ANOVA with Tukey's HSD post hoc test). (**D**) Schematic of the Opto-thermocycler assay. (**E**) Percent of mosquitoes flying in response to 60°C heat for the indicated genotypes (n = 7-8 trials/genotype). (**F**) Quantification of mosquitoes flying during each experimental trial before and after 1 minute after a 4-second pulse of 60°C heat for the indicated genotypes (n = 7-8 trials/genotype, $p > 0.05$, between all genotypes, post 60°C heat, one-way ANOVA with Tukey's HSD post hoc test).

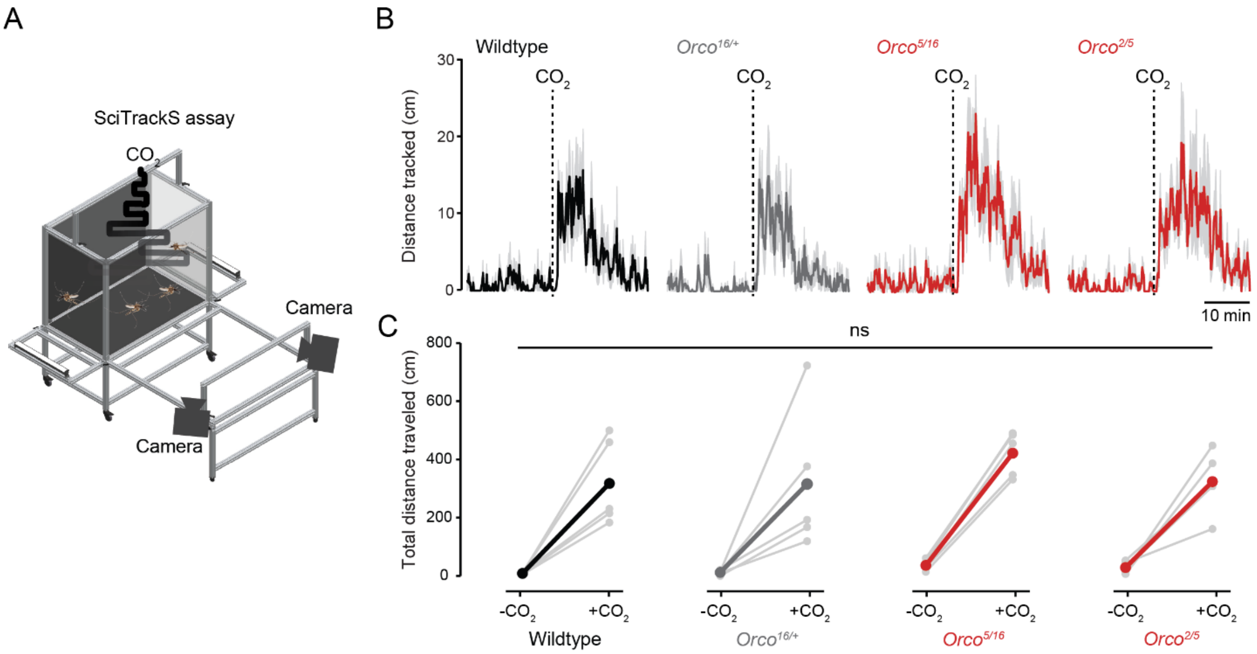

Fig. S2. – Related to Fig. 3

***Orco* mutants display normal CO_2-evoked activity.**

(**A**) Schematic of the SciTrackS assay. (**B**) Population distance flown in response to stimulation with CO_2 for the indicated genotypes (mean ± SEM, 10-second bins, n = 5 trials, 20 animals/trial). (**C**) Cumulative distance tracked per mosquito in the 6 minutes immediately before or after CO_2 addition (n = 5 trials/genotype, $p > 0.05$, between all genotypes post CO2, one-way ANOVA with Tukey's HSD post hoc test).

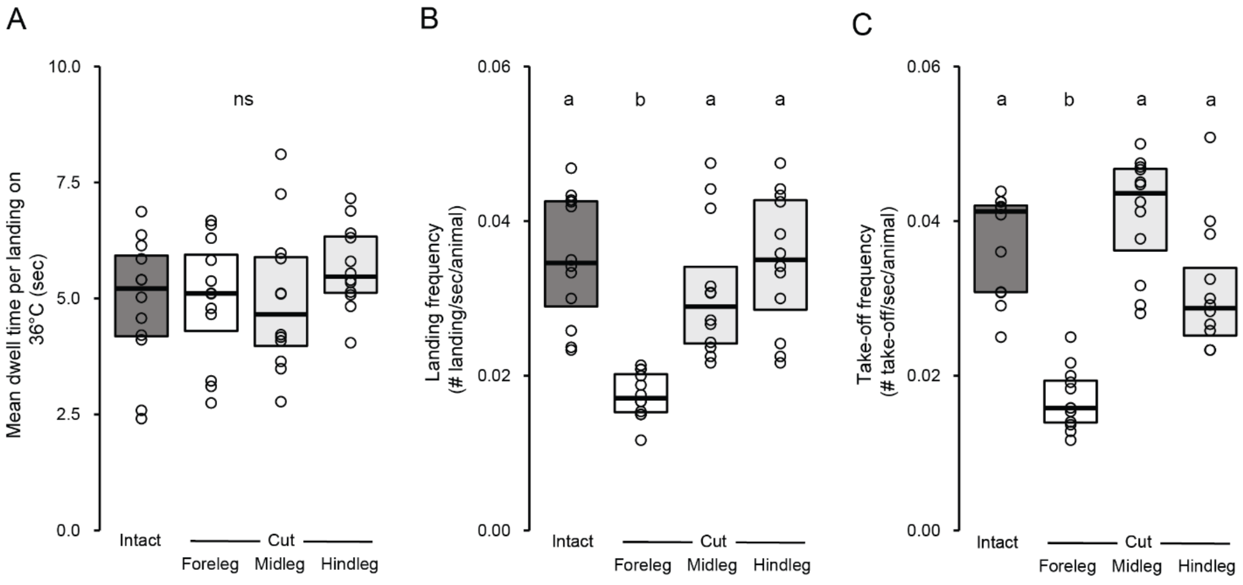

Fig. S3. – Related to Fig. 4
Removing the mosquito foreleg tip impacts heat-seeking behavior.
(**A**- **C**) Mean dwell time (**A**), landing frequency (**B**), and take-off frequency (**C**) of indicated tarsal treatment on the Peltier surface during the 36°C trial (n = 9 trials/condition). Data are plotted as scatter-box plots (individual data points, median as horizontal line, interquartile range as box). Data labeled with different letters differ significantly ($p < 0.05$; one-way ANOVA with Tukey's HSD post hoc test).

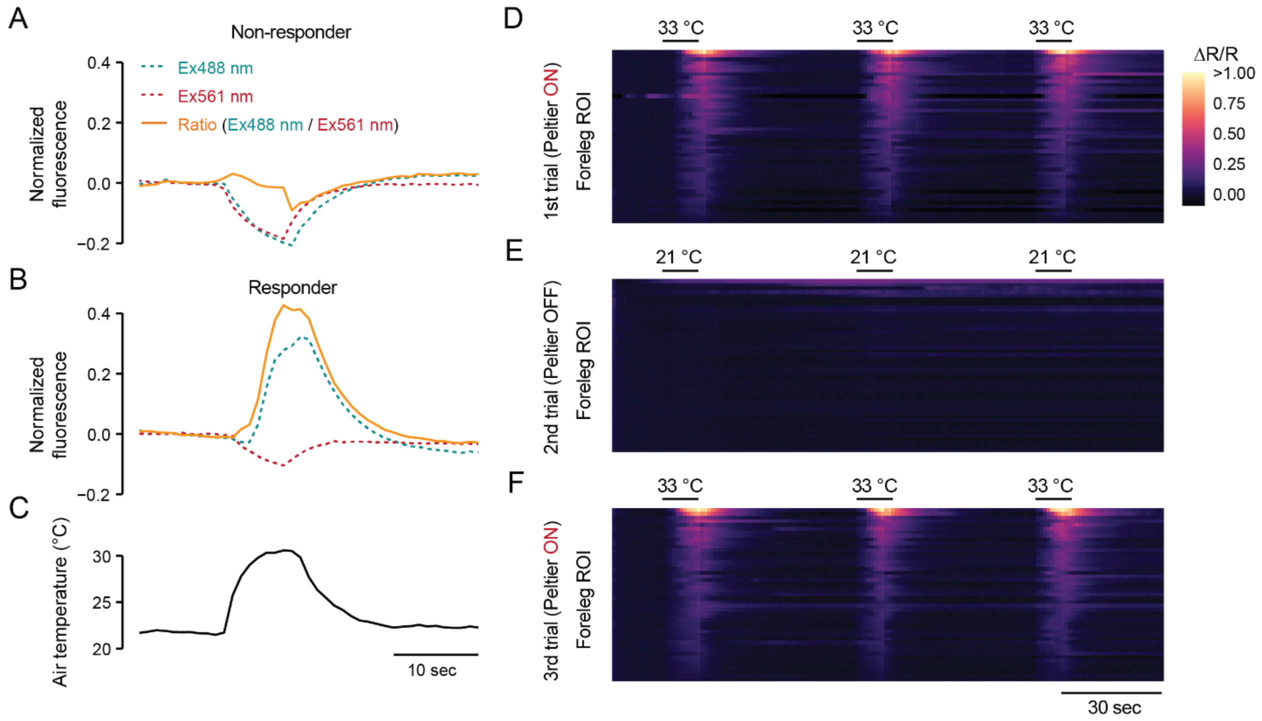

Fig. S4. – Related to Fig. 6

Ratiometric Ca^{2+} imaging analysis makes it possible to distinguish non-responders from responders.

(**A-C**) Representative fluorescent traces from green, red, and ratiometric channels were acquired from a non-responding (**A**) and a responding cell (**B**) in response to a heat pulse (**C**). (**D-F**) Fluorescent signals from foreleg neurons across three heat pulse trials, each consisting of three consecutive pulses of heat stimuli. The first (**D**) and third (**F**) trials had the Peltier element on, while the second was turned off (**E**). n = 47 ROI.

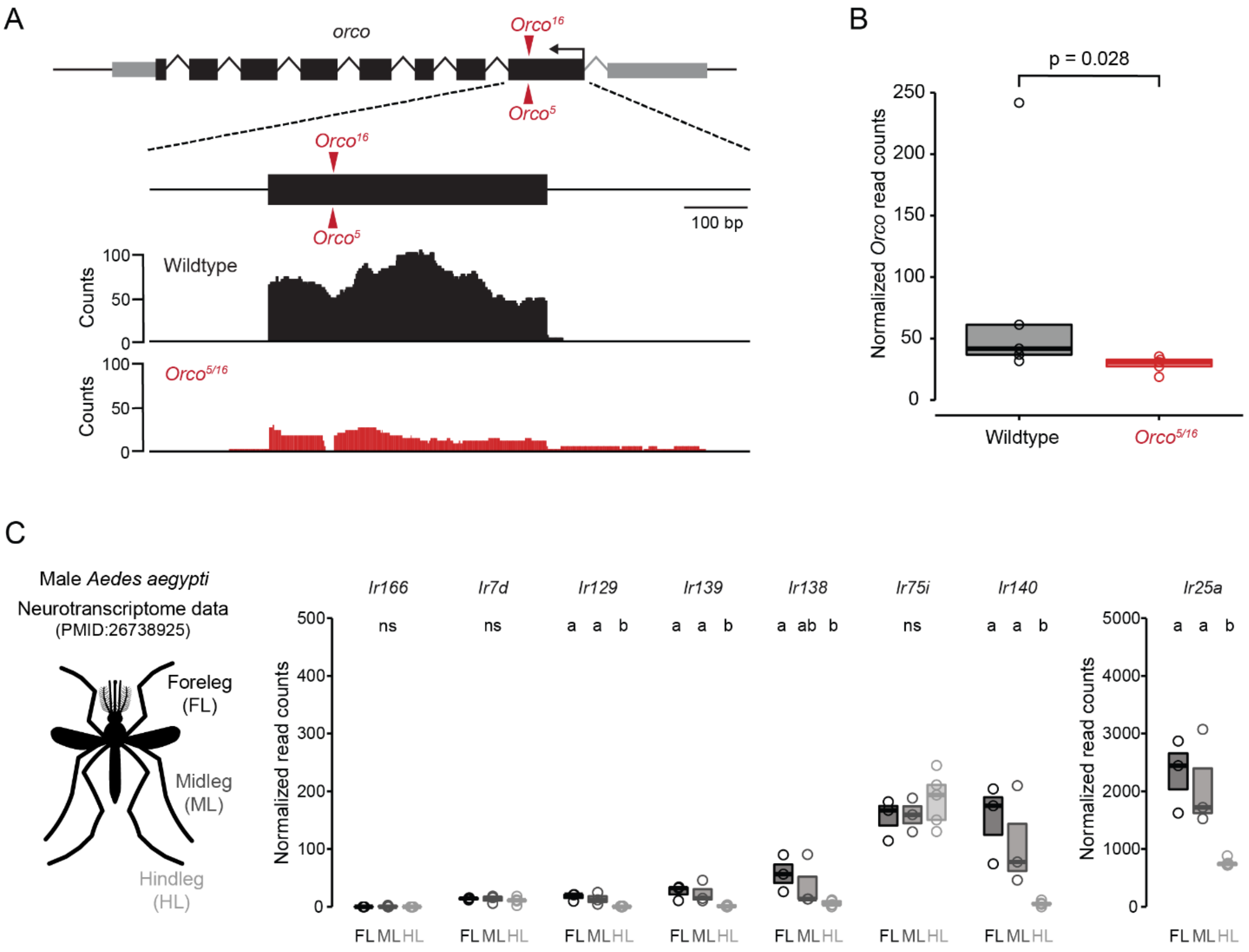

Fig. S5. – Related to Fig. 8

***Aedes aegypti* leg transcriptome analysis shows reduced Orco RNA in *Orco* mutants and differential expression in the different pairs of legs.**

(**A**) Graphical representation of the number of raw mapped reads at the first exon at the *Orco* locus from wildtype (black) and $Orco^{5/16}$ mutant leg RNAseq dataset (red). $Orco^{5}$ and $Orco^{16}$ loci are indicated with red arrowheads. (**B**) Normalized *Orco* read counts from wildtype and $Orco^{5/16}$ legs (DESeq2, FDR < 0.05). (**C**) Normalized read counts of $Orco^{5/16}$ leg upregulated IR genes in a previously published male leg neurotranscriptome dataset (*30*). Data labeled with different letters are significantly different (n=3-5 replicates/leg, FDR < 0.01, pairwise DESeq2 comparison). Data are plotted as scatter-box plots (individual data points, median as horizontal line, interquartile range as box) for (**B**, **C**).

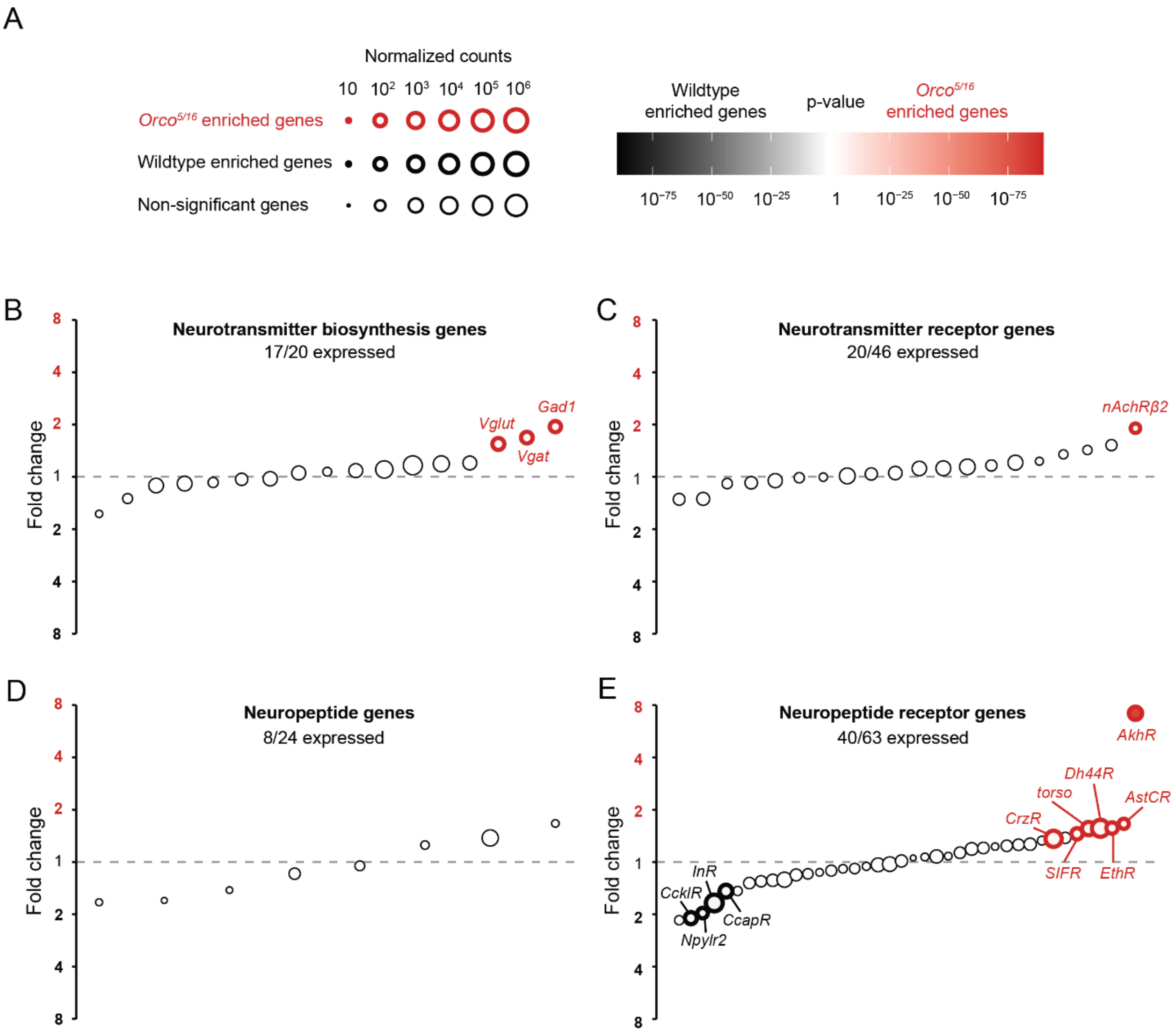

Fig. S6. – Related to Fig. 8

***Aedes aegypti* neuromodulatory gene expression changes in the *Orco* mutant legs.**

(**A**) Legend for bulk RNA-seq data in panels B-E. (**B**-**E**) Gene expression comparisons between wildtype and *Orco*$^{5/16}$ mutant mosquito tarsi for neurotransmitter biosynthesis genes (**B**), neurotransmitter genes (**C**), neuropeptides (**D**), and neuropeptide receptors (**E**). Genes are listed from left to right from lowest to highest fold-change enrichment in *Orco*$^{5/16}$ tarsi compared to wildtype. Y-axis is represented as fold change on a $\log_2$-scale.

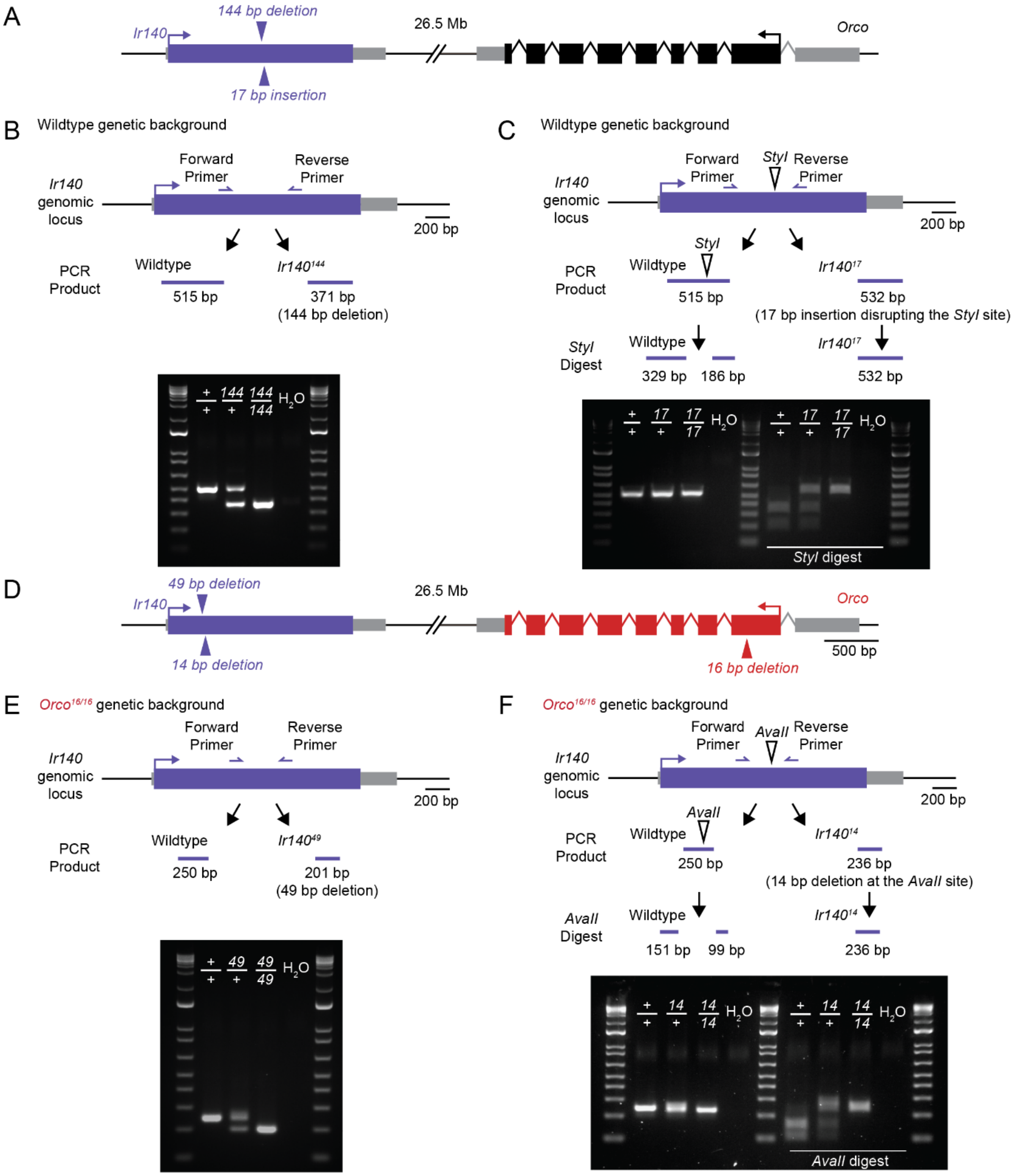

Fig. S7. – Related to Fig. 9

PCR genotyping was used to confirm *Ir140* single and *Orco, Ir140* double mutant strains. (**A**, **D**) Schematic of the *Ir140*, *Orco* loci, and the isolated alleles for single (**A**) and double mutants (**D**). (**B**, **C**, **E**, **F**) Genotyping schematics (top) and agarose gel electrophoresis images (bottom) of PCR fragments. PCR fragments before (left) and after (right) restriction digests with the indicated restriction enzymes to genotype the indicated *Ir140* (**C**) and *Orco, Ir140* double mutants (**F**).

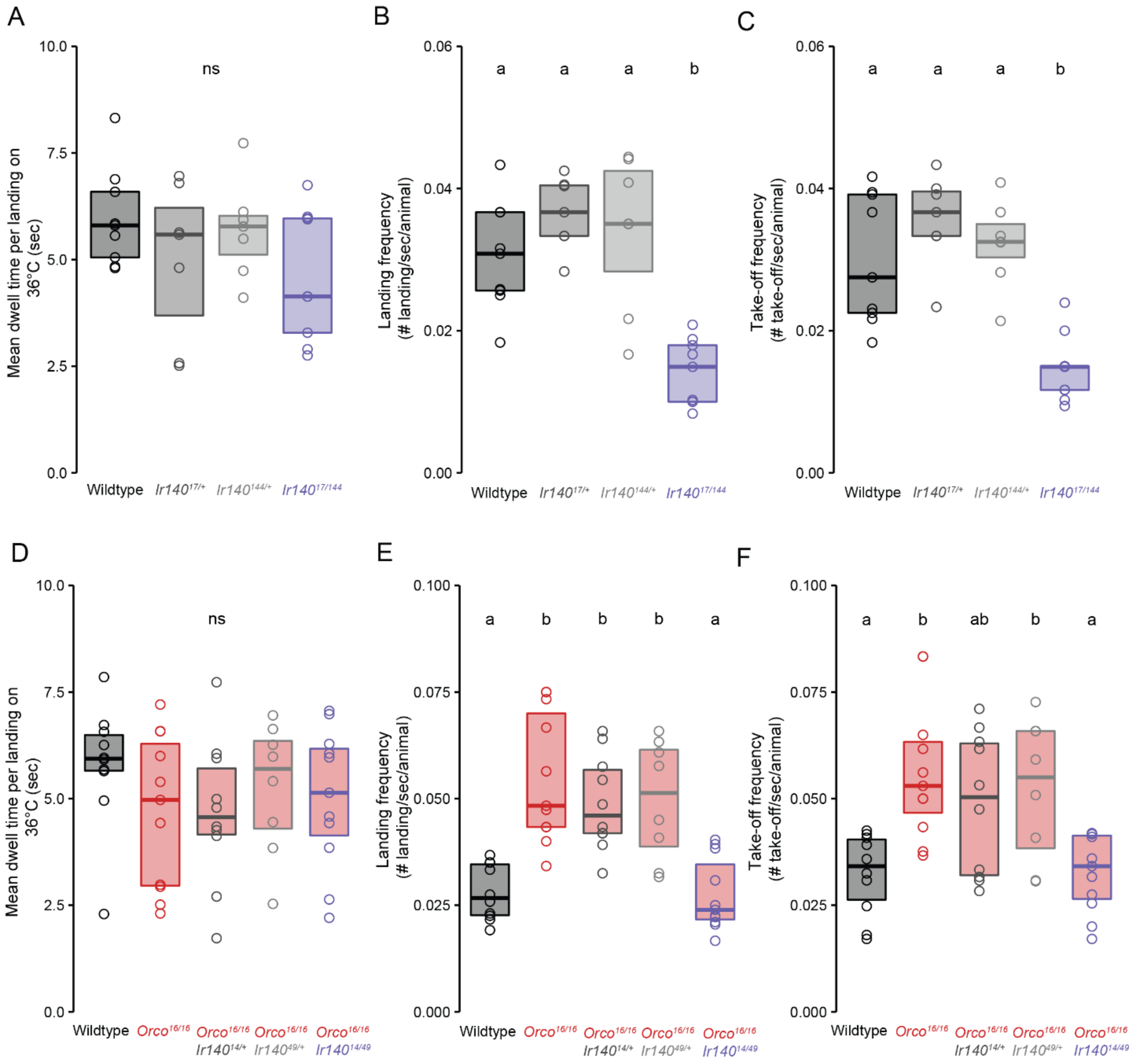

Fig. S8. – Related to Fig. 9

***Ir140* mediates heat-seeking behavior.**

(**A**- **F**) Mean dwell time (**A, D**), landing frequency (**B, E**), and take-off frequency (**C, F**) of indicated genotype on the Peltier surface during the 36°C trial (n = 7-11 trials/genotype). Data are plotted as scatter-box plots (individual data points, median as horizontal line, interquartile range as box). Data labeled with different letters differ significantly ($p < 0.05$; one-way ANOVA with Tukey's HSD post hoc test).

Movie S1 – Related to Fig. 1
Mosquitoes are attracted to a heated Peltier.
Percent mosquitoes on Peltier (top left) quantified using images (right) acquired across the 36°C heat stimulus (bottom left). CO_2 pulse is indicated in green, and the Peltier location is outlined with a dotted line. The images are acquired at 1 Hz, and the movie is sped up 20X.

Movie S2 – Related to Fig. 2
***Orco$^{5/16}$* mutant mosquitoes show increased attraction to heat.**
Heat-seeking behavior comparing wildtype control (middle) and *Orco$^{5/16}$* mutants (right) during the 36°C heat stimulus (left). CO_2 pulse is indicated in green, and the Peltier location is outlined with a dotted line. The images are acquired at 1 Hz, and the movie is sped up 20X.

Movie S3 – Related to Fig. 5
Tarsal neurons are differentially distributed along the cranial-caudal axis.
3D reconstruction of dTomato confocal image of forelegs taken from a *brp-QF2w* > *QUAS-dTomato-T2A-GCaMP6s* animal.

Movie S4 – Related to Fig. 6
Ratiometric calcium imaging of foreleg tarsal neurons shows responses of neurons to heated air.
An example *ex vivo* foreleg tarsal imaging experiment with three consecutive heat pulses. The right panel is a movie of a ratiometric image of the three most distal tarsal segments. The foreleg is outlined with a white dotted line. The top right panel shows the ratiometric fluorescent signals across the experiment. Each trace indicates ratiometric calcium responses from regions highlighted in the same color as the right panel. The blue trace represents a non-responding cell. The bottom right panel shows the temperature traces. White arrowheads indicate heat pulses.
